# Magnesium-Based Temporary Implants: Potential, Current Status, Applications, and Challenges

**DOI:** 10.3390/jfb14060324

**Published:** 2023-06-17

**Authors:** Sankaranarayanan Seetharaman, Dhivya Sankaranarayanan, Manoj Gupta

**Affiliations:** 1 Department of Mechanical Engineering, College of Design and Engineering, National University of Singapore, 9 Engineering Drive 1, Block EA #07-08, Singapore 117575, Singapore; ssnseetharaman@yahoo.com (S.S.); sndhivya@nus.edu.sg (D.S.); 2 Advanced Remanufacturing and Technology Centre (ARTC), Agency for Science, Technology and Research (A*STAR), 3 Cleantech Loop, #01/01 CleanTech Two, Singapore 637143, Singapore

**Keywords:** magnesium alloys, implants, biodegradable, biocompatible, degradation behavior, applications, commercial implants, clinical trials

## Abstract

Biomedical implants are important devices used for the repair or replacement of damaged or diseased tissues or organs. The success of implantation depends on various factors, such as mechanical properties, biocompatibility, and biodegradability of the materials used. Recently, magnesium (Mg)-based materials have emerged as a promising class of temporary implants due to their remarkable properties, such as strength, biocompatibility, biodegradability, and bioactivity. This review article aims to provide a comprehensive overview of current research works summarizing the above-mentioned properties of Mg-based materials for use as temporary implants. The key findings from in-vitro, in-vivo, and clinical trials are also discussed. Further, the potential applications of Mg-based implants and the applicable fabrication methods are also reviewed.

## 1. Introduction

Implants refer to medical devices that are used to replace or augment a part of the body. As they enable the repair and replacement of damaged tissues and organs, they are used for a wide range of applications including orthopedics, cardiovascular, and dental procedures. The key characteristics of implants include biocompatibility, mechanical strength, stability, and functionality [1,2]. Biocompatibility refers to the ability of implant to interact with the surrounding tissue without causing an adverse reaction or rejection. Therefore, implants must be made from non-toxic and non-reactive materials. The mechanical strength must be adequate to withstand the stresses and strains of daily activities, especially when considered for long-term use. For stability, the implants must be securely anchored to the surrounding tissue or bone to prevent any movement or instability. With respect to functionality, the implants must be capable of performing the intended function such as providing support, enhancing mobility, or improving overall health and well-being [3,4].

Based on the intended period of usage, there are mainly two types of implants: permanent and temporary. Permanent implants are designed to remain in the body for the rest of the patient’s life. They are typically made from materials, such as titanium or stainless steel, which are known for their strength, durability, and biocompatibility. These materials are inert, meaning that they do not react with the surrounding tissue and are not absorbed by the body. As a result, permanent implants are often used in applications where long-term stability and durability are required. Temporary implants, on the other hand, are designed to be either removed from the body after a short period of use or be gradually absorbed by the body over time once the healing process in complete. They are typically used for applications where the body will naturally heal itself over time, such as in orthopedic procedures or in certain cardiovascular applications. Temporary implants are made from a variety of materials, including polymers, ceramics, and metals. The choice of material will depend on the specific application, as each material has its own set of properties and characteristics [5,6].

When it comes to temporary implants, biodegradable materials have garnered significant attention in recent years [6,7]. One such material is magnesium, a biodegradable metal with a unique combination of properties that makes it an attractive option for temporary implant applications. Magnesium (Mg) is a lightweight metal with a density similar to that of the human bone. Mg is also biocompatible, meaning that it does not cause any harmful immune response or other adverse reactions in the body and hence can be safely used in medical applications. Unlike permanent implants made of materials like titanium or stainless steel, which tend to remain unaffected in the body for a long time [8], Mg has the ability to degrade naturally in the body by breaking down into harmless magnesium ions that can be metabolized and excreted [9]. This helps to eliminate the need for surgical removal of implants once the purpose is served, which hence reduces the risk of complications and overall treatment cost. Some of the recent literature also highlights the tendency of Mg ions to actively enhance the proliferation and differentiation of bone-forming cells, thereby stimulating the formation of new bone tissue [10].

In addition to its interesting biological attributes, Mg also has the unique combination of mechanical properties which are similar to those of the natural bone [11]. Its low modulus of elasticity reduces the risk of implant rejection or failure caused by stress shielding due to a larger mismatch in the elastic moduli of bone and implants [12,13]. This highlights the fact that Mg implants can mimic the shape and structure of bone, if designed carefully, and hence exhibit a reduced risk of implant rejection or failure [14]. Despite the potential of magnesium for temporary implant applications, there are some challenges associated with using this material. One of the main challenges is the potential for rapid corrosion in the harsh environment of the body. This can lead to premature degradation of the implant and the release of potentially toxic by-products. In this regard, current research works in this field explore novel alloys and a variety of surface treatments methods used to slow down the corrosion rate to match the rate of tissue regeneration [15,16].

In view of the benefits as stated above, several extensive reviews have been carried out in the past highlighting the mechanical and biocorrosion attributes of Mg alloys [17,18,19,20,21,22]. However, there is still a need for systematic and consolidated reviews on the state of the art, applications, and challenges of Mg-based temporary implants. Hence, an attempt in made in this review article to summarize the mechanical and biocorrosion attributes of several Mg-based materials; the outcomes of various in vivo, in vitro, and clinical trials; and current applications, fabrication methods, and current challenges applicable for Mg-based temporary implants.

## 2. Desired Characteristics of Implants

Implants refer to medical devices or objects that are surgically placed inside the body to serve a variety of purposes, such as restoring mobility, regulating bodily functions, or monitoring certain aspects of the body [2]. The common examples include pacemakers, artificial joints, dental implants, cochlear hearing aids, dermal fillers, breast implants, etc. [23]. They can be made from a variety of materials including metals, polymers, ceramics, or biological materials, depending on the intended use and specific requirements [24]. In general, the implants must meet the following criteria to ensure that they are safe and effective when inserted into the body. Each of these features is crucial for the safety, efficacy, and longevity of body implants and they are often interdependent. Therefore, implants undergo rigorous testing and clinical trials before being approved for the intended applications [25,26].

1. Similar Density and Young’s Modulus as Surrounding Tissues: Implants should have similar or comparable density and Young’s modulus as that of the surrounding tissues. Any significant differences will create stress concentrations leading to potential implant failure, inflammation, tissue damage, and bone loss. For example, the dental implant will experience excessive stress during chewing when it has a higher Young’s modulus than the surrounding bone. Hence, the careful selection of implant materials with comparable density, and Young’s modulus is crucial to ensure the proper and safe functioning of implants without stress field effects.
2. Adequate mechanical strength: It is a crucial property for load bearing implants like orthopedic and dental implants, as they are designed to replace or augment bones or joints. In general, the implants are expected to be strong enough to withstand the forces and stresses that it will encounter within the body, without compromising its structural integrity. For example, the dental implants are specifically designed to handle mechanical loads from biting and chewing of foods. Normally, the mechanical strength requirements are specified in terms of tensile, compressive, or bending strength, and their typical values range from 50 to 2000 MPa for tensile strength, 100 to 2000 MPa for compressive strength, and 50 to 1000 MPa for bending strength.
3. Corrosion resistant: It is particularly important for metallic implants that are in contact with body fluids as some metals tend to corrode upon exposure to body fluids like blood, plasma, or intestinal fluid, which may then lead to the loss of structural integrity, failure, toxicity, or other complications. Hence, it is essential to control the corrosion of permanent implants by choosing the right material that remains inert in body fluids (e.g., titanium or stainless steel) or by using protective coatings. The typical values for corrosion resistance for body implants range from 0.1 to 10 mpy (mils per year) as measured by corrosion tests such as ASTM F2129.
4. Biocompatible: One of the major requirements for body implants is biocompatibility as incompatible implants can cause infection, inflammation, rejection, and other complications. Hence, the implant materials are specifically designed to coexist with the living tissues without causing any adverse reactions or immune response in the body. The biocompatibility of implants can be evaluated by test procedures like MTT (3-(4,5-dimethylthazolk-2-yl)-2,5-diphenyl tetrazolium bromide) assay, cell adhesion, cell proliferation, alkaline phosphatase (ALP) activity, and compatibility tests as per ISO 10993 [27].
5. Radiopacity: This is the ability of the implant to be visible on medical imaging devices such as X-rays and CT scans. It is a crucial factor while selecting materials for implants as it enables easy detection on X-rays and accurate diagnosis for treatment planning.
6. Ability to withstand sterilization: Implants must be sterilized before implantation to ensure that it is free from harmful microorganisms to prevent infections and other complications. Therefore, it is essential for implants to withstand sterilization methods such as autoclaving, gamma radiation, ethylene oxide gas, or other chemical treatments.
7. Biodegradability and bioresorbable nature: Implants can also be designed to gradually break down and be absorbed by the body over time, as in the case of biodegradable implants, with the degradation products being either metabolized or excreted. Such implants can be made from materials that the body can absorb and integrate into surrounding tissue, e.g., synthetic or natural polymers, such as polylactic acid (PLA) or collagen. They offer potential advantages, such as reduced risk of complications and improved healing times, as well as a potential reduction to treatment cost.

Based on the intended period of application, implants can be classified into either permanent or temporary implants [1], and the key differences lie in their intended use, duration of placement, design considerations, and materials, as listed in Table 1.

One of the major limitations of temporary implants is the need for multiple surgeries. Unlike permanent implants that are designed to remain in the body for an extended period, temporary implants have a limited lifespan and need to be removed after a specific period, which means that patients may need to undergo multiple surgeries to implant and remove the device. To address this limitation, novel implants are being designed to remain in the body for a specific period followed by their natural degradation and absorption upon completion of their useful life. These implants are typically made of materials, such as polylactic acid (PLA), polyglycolic acid (PGA), or collagen, that are biocompatible, biodegradable, and bioresorbable, and they have a wide range of applications in different medical fields [28]. This eliminates the need for a corrective surgery to remove the implant once it is no longer needed and therefore simplifies the recovery process for the patient by reducing the risk of complications associated with implant removal, such as infection and bleeding, as well as reducing the overall cost of treatment.

In general, the biodegradable and bioresorbable temporary implants (e.g., PLA and PGA) are used in orthopedic applications to repair and regenerate bone tissue. They tend to stabilize fractures, support damaged bones, and promote bone regeneration. It also stimulates the growth of new bone tissue by releasing the biologically active compounds upon degradation. Similarly, in cardiology, biodegradable stents made from polycaprolactone (PCL) and poly-l-lactic acid (PLLA) are used to treat coronary artery disease. While this class of temporary implants made of polymers present unique advantages, as mentioned earlier, they have a shorter lifespan compared to permanent implants and may not be suitable for load-bearing applications such as in orthopedics. Furthermore, there is also a risk associated with the degradation process that is difficult to predict as the degradation of implant depends on various factors including implant chemistry, patient physiology, location of the implant, etc. [28,29,30]. In this regard, metallic magnesium shows great promise as a material for implant applications due to its unique combination of mechanical strength similar to that of natural bone, biocompatibility, and biodegradability. As a biodegradable metal, magnesium tends to gradually degrade and get absorbed by the body. It not only eliminates the need for a second surgery for implant removal, but also promotes tissue regeneration [7,31,32].

## 3. Magnesium for Temporary Implants

**Figure 1:**
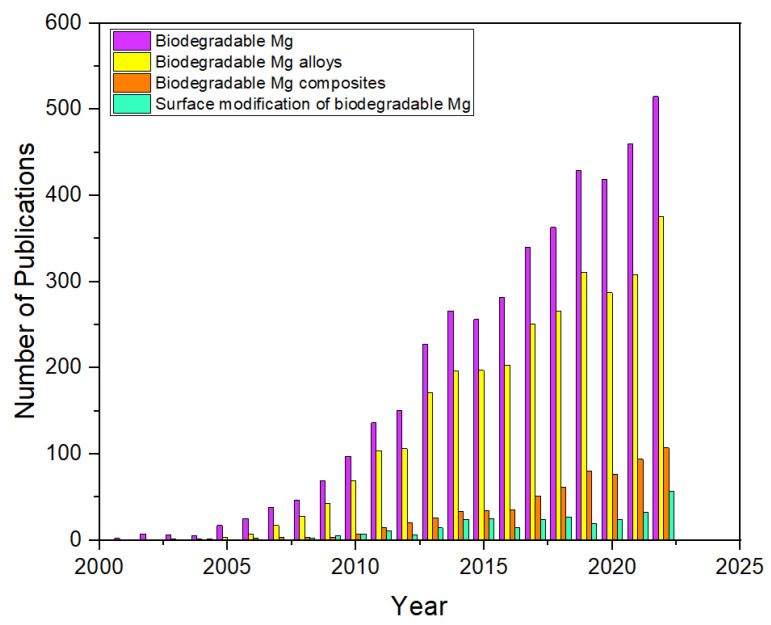
Publication history on biodegradable implants focused on Mg-based implants and coatings. (Source Data from Scopus Search).

Magnesium-based implants have emerged as a promising alternative to traditional metallic implants. They have a rich history since the early 20th century, beginning with their use in dental applications [6]. While the uncontrolled degradation of Mg in body fluids limited its extended use for many years, research advances in terms of novel alloys and surface treatment methods have made it a promising alternative to traditional permanent implants. Currently, Mg implants are being explored for several applications including bone fixation, cardiovascular stents, and drug delivery systems, and the ongoing research efforts are very much focused on perfectly controlling and predicting the degradation rates [28]. Figure 1 shows the increasing number of research publications in the field of biodegradable and magnesium implants in the last two decades.

(i) Mechanical Properties

In general, the mechanical properties of Mg alloys can be tailored by adding selective alloying elements [33,34]. For example, Aluminum (Al) and Zinc (Zn) are the most common alloying elements of Mg, and their combined addition generally improves the strength and overcomes the harmful corrosive effects of iron and nickel impurities. While a minimum of 6% Al is required to induce precipitation hardening benefits during heat treatment, the Zn content has to be properly controlled below 5% to prevent hot shortness. The addition of Zn in combination with Zirconium (Zr) and rare earth (RE) metals also yields high strength by precipitation hardening, as Zr is an effective grain refiner for Mg with similar lattice parameters (aZr = 0.323 nm, cZr = 0.514 nm, aMg = 0.320 nm, cMg = 0.520 nm). On the other hand, the combined addition of Zr and Al is not recommended because of their tendencies to form stable phases. In general, the ZE series alloys have better high-temperature properties and corrosion resistance compared to AZ alloys, as RE addition in general is known to improve the ductility, high temperature creep resistance, and corrosion resistance of Mg, as well as facilitate the elimination of porosities in cast Mg alloys as they narrow down the metal freezing range of the alloys. In particular, yttrium with high solid solubility in Mg (12.4%) improves the creep resistance, thermal stability, corrosion resistance, and deformation behavior (better ductility and work hardening) of wrought Mg–Y alloys when added together with other RE elements such as cerium or neodymium. Calcium (Ca) addition is also beneficial for light weighting and oxidation control. While Ca improves rollability of Mg sheets, excess calcium leads to cracking during the welding process. It is also beneficial for corrosion resistance as they tend to form a protective calcium phosphate layer on the surface of the material. Ca addition is also being popularly explored for developing biomedical alloys as Ca being a major component of the bone. Similarly, silver additions also are being explored for potential antimicrobial benefits. Ag can also improve the mechanical properties and corrosion resistance by age hardening [35,36,37]. It is important to note that researchers are attempting to avoid the use of aluminum and rare earth elements in developing Mg-based materials for bioapplications due to their toxic effects.

In this regard, different reinforcements have also been used to improve the mechanical properties of Mg alloys for applications as temporary implants. Conventionally, biocompatible oxides, such as alumina (Al_2_O_3_), zirconia (ZrO_2_), silica (SiO_2_), titania (TiO_2_), and zinc oxide (ZnO), are used as reinforcement to develop Mg composites for biomedical applications. Al_2_O_3_ is a bioinert ceramic that can improve the hardness, strength, and abrasion resistance of Mg and is therefore ideal to develop Mg-based metal matrix composites for load-bearing applications such as orthopedic implants like bone screws, plates, and knee prosthetics. The non-toxic and biochemically inert ZrO_2_ also offers similar benefits. In this regard, TiO_2_ also improves the mechanical properties of Mg and offers least cytotoxicity compared to other metal oxides [38,39,40].

Recent research works also highlight the potential use of several biodegradable ceramic and polymers as reinforcement to develop Mg implants. This includes examples such as polylactic acid (PLA), polyglycolic acid (PGA), hydroxyapatite (HA), tricalcium phosphate (TCP), etc. PLA and PGA are the biodegradable polymers that can degrade in the body over time. Although their influence on mechanical properties is relatively insignificant, they are found to enhance bone regeneration and integration with the surrounding tissue and hence used as reinforcement in Mg implants. HA is a biocompatible calcium phosphate ceramic with chemical and crystal structure similar to the mineral component of bones and teeth. It enhances the mechanical properties of Mg, such as hardness and compressive strength, making it more suitable for load-bearing applications. TCP is another calcium phosphate ceramic that is extensively used as a reinforcement owing to its biodegradable and bioresorbable characteristics. Similar to HA, TCP also improves the mechanical properties of Mg. Silica bioglass ceramic has also been used as a reinforcement in Mg implants owing to its bioinertness and its ability to improve the mechanical properties [38,39,40,41,42].

**Figure 2:**
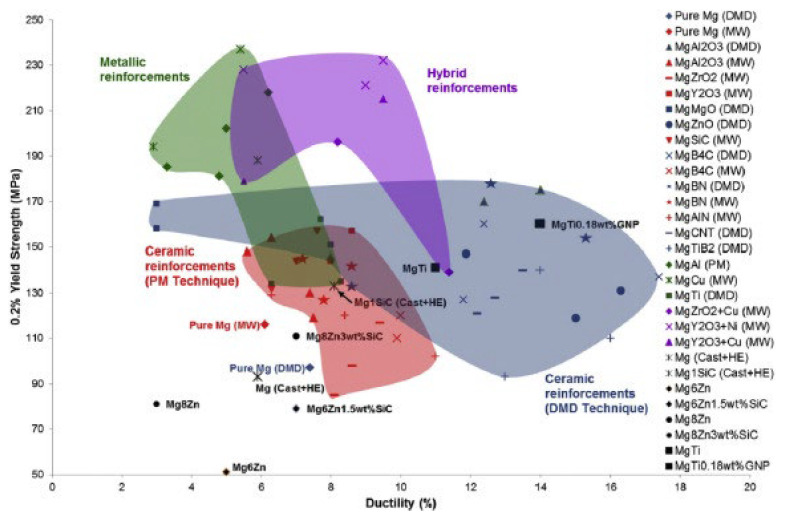
Mechanical strength of magnesium-based nanocomposites (Source: Song et al. [45]).

Table 2 lists the mechanical properties of the few Mg alloys and composites that are potentially suitable for biomedical applications. Compared to traditional reinforcements, the larger surface to volume ratio of the nanoparticles induces significant strengthening, as shown in Figure 2. Hence, the addition of nano-length scale reinforcements is carried out in smaller quantities, usually less than 5 vol%. Further, it is worth noting that the strength improvement in most cases occurred without any adverse effects on ductility and fracture toughness [43,44].

The above table (Table 2) also compares the mechanical properties and corrosion resistance of selected biomedical Mg alloys with other traditional permanent and temporary implant materials. When compared to conventional implant materials, Mg-based materials exhibit several unique properties that make them suitable for temporary implant applications.

- Tensile strength: Magnesium-based materials have a lower tensile strength than titanium and stainless steel, but their strength-to-weight ratio is higher, which makes them an attractive option for lightweight implant applications. In this regard, it is also worth noting that the newly developed Mg alloys and composites have strength and ductility comfortably exceeding that of cortical bone.
- Elastic modulus: The elastic modulus of Mg alloys and composites is relatively low compared to titanium and stainless steel, which helps to minimize stress-shielding effects and prevent bone resorption that can occur with stiffer implant materials.
- Ductility: Magnesium alloys have relatively poor ductility compared to many metallic biomaterials, including titanium and stainless steel. For these reasons, novel Mg alloys are being developed with superior plastic deformation capabilities for load-bearing applications.
- Fatigue strength: The fatigue strength of magnesium-based materials is typically lower than that of titanium and stainless steel, which may limit their use in applications with higher cyclic loading.
- Corrosion resistance: While Mg has a lower corrosion resistance than titanium and stainless steel, its biodegradability can be an advantage in temporary implant applications. Similarly, compared to polymer and ceramic implant materials, Mg exhibits higher strength, better ductility, and a lower modulus of elasticity. These properties make Mg highly suitable for load-bearing applications.

(ii) Biocompatibility

While Mg has similar mechanical properties as human bones and can be gradually absorbed by the body, thereby eliminating the need for surgical removal, its usage as implants is still limited due to the inherent poor corrosion resistance and biocompatibility. In this regard, alloying additions can improve these properties to make them highly suitable for temporary implant applications. The general effects of relevant alloying additions and reinforcements on the biocompatibility of Mg are summarized in Table 3 [35,36,37].

- Manganese and lithium are shown to be beneficial for anti-inflammatory properties.
- Reinforcement: Naturally occurring calcium phosphate mineral ceramics like tricalcium phosphate and hydroxyapatite have been used as reinforcement to develop Mg composites for biomedical applications. Being a major component of bone and teeth, they are biocompatible and well-tolerated by the human body. Similar benefits were also reported when synthetic silica-based bioglass ceramic was used as a reinforcement in Mg. Biodegradable polymers, such as polylactic acid (PLA) and polyglycolic acid (PGA), are also used as biodegradable reinforcements as they can be metabolized into lactic and glycolic acids that are naturally occurring in the body [38,39,40].

(iii) Biodegradation

**Figure 3:**
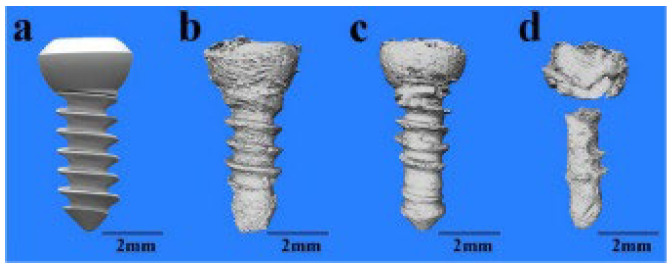
Biodegradation of Mg screw as visualized by X-ray synchrotron radiation: (**a**) initial state; (**b**) 3D images reconstructed after 1 month from implantation, (**c**) after 4 months from implantation, and (**d**) after 7 months from implantation (Source: Tsakiris et al. [31] open access publication.)

Biodegradation refers to the process by which a material breaks down over time in the physiological environment and gets metabolized or excreted by the body [46]. Figure 3 shows the biodegradation of a Mg screw as visualized by X-ray synchrotron radiation. The major advantage of using Mg-based implants is that they are biocompatible and biodegradable, thereby eliminating the need for implant removal surgery. Moreover, the biodegradation products of Mg-based implants also have a positive effect on bone growth as Mg ions can be readily metabolized and excreted by the body. However, the disadvantage of using Mg-based implants is that their mechanical properties can degrade over time, leading to a decrease in their functional lifetime. Moreover, the corrosion products of Mg-based implants can cause an inflammatory response, leading to adverse tissue reactions [31].

The biodegradation of Mg implants occurs in several stages, including surface oxidation, corrosion, and dissolution [47,48].

Surface oxidation: It is the first stage in the degradation process where the Mg-based implants, upon exposure to biological environments, develop a thin oxide layer due to the reaction with oxygen and water molecules in the body fluids. This oxide layer tends to serve as a protective layer and prevents the further corrosion and degradation of the Mg implant.

Corrosion: In the second stage, the Mg implant starts to corrode actively in the biological medium by electrochemical reaction with implants acting as the anode and the body fluids as the electrolyte. The mechanism can be explained using the following electrochemical reactions. In general, the evolution of H_2_ gas and the protective layer formation are expected to decline the rate of cathodic reaction. However, the weak protective layers tend to break down before anodic polarization, thereby resulting in faster degradation.

$$\mathrm{Mg}\;\to {\;\mathrm{Mg}}^{2+}+2e^{-}\;\mathrm{(i) Anodic reaction}$$

$$2H_{2}O+2e^{-}\to {\;H}_{2}+2{\mathrm{OH}}^{-}\;\mathrm{(ii) Cathodic reaction}$$

$${\mathrm{Mg}}^{2+}+2{\mathrm{OH}}^{-}\to \;\mathrm{Mg}{\left(\mathrm{OH}\right)}_{2}\;\mathrm{(iii) Product formation}$$

Though the above reactions are universal for Mg corrosion in aqueous media, the presence of dissolved oxygen, proteins, amino acids, chloride, and hydroxide ions tend to affect the degradation mechanisms. In general, the Mg^2+^ ions resulting from the biocorrosion process will combine with the anions, such as chloride or sulphate, to form magnesium salts as shown below.

$$\mathrm{Mg}{\left(\mathrm{OH}\right)}_{2}+2{\mathrm{Cl}}^{-}\to {\;\mathrm{MgCl}}_{2\;}+2{\mathrm{OH}}^{-}$$

$${\mathrm{Mg}}^{2+}+2{\mathrm{Cl}}^{-}\to {\;\mathrm{MgCl}}_{2}$$

With respect to the corrosion process, while the adsorption of amino acids, proteins, and lipids present in the physiological over the surface of Mg implant affect its degradation rate, the high concentration of chloride ions promotes pitting corrosion by breaking down the protective Mg(OH)_2_ hydroxide layer before anodic polarization.

Dissolution: In the final stage, the Mg implant is gradually dissolved by the body fluids, and the released Mg ions are transported away through the body fluids. They are then utilized by the body for various metabolic processes, including bone formation, protein synthesis, and DNA synthesis, and the excess ions along with other corrosion products are finally excreted through the urinary system.

In general, the degradation process leads to a decrease in the mechanical integrity of the implant, resulting in pores or cracks that act as sites for localized corrosion to further accelerate the degradation process. Hence, understanding the mechanism of biodegradation is crucial for developing biodegradable Mg implants with desirable properties for specific applications. The biodegradation rate of Mg-based implants can be controlled by various methods, including the addition of alloying elements and surface modification. In general, the addition of alloying elements, such as Al, Mn, Zn, Zr, Ca, and rare earth elements enhances the corrosion resistance of Mg-based implants in aqueous media, and they can be effectively used to control the biodegradation rate. However, there are known issues associated with the biocompatibility due to their excess addition. In particular, Al, which is a major alloying addition for Mg, has been implicated as a potential risk factor in Alzheimer’s disease [35,37].

The surface modification approach can also be used to tailor the properties of the implant’s surface to control the biodegradation rate of Mg implants. One of the most common methods is to apply biocompatible coatings on the implant’s surface to prevent accelerated corrosion. For example, hydroxyapatite (HA) and calcium phosphate (CaP) coatings have been used to improve corrosion resistance of Mg alloys without affecting the biocompatibility [49,50]. These coating materials are known to promote bone growth and implant’s integration with the surrounding tissue. Another method of surface modification is surface roughening, which helps to enhance cell adhesion and promote tissue growth by increasing the effective surface area of implants. However, excessive surface roughness can also lead to increased corrosion rates. Therefore, the optimal surface roughness should be carefully controlled to balance the benefits of enhanced biocompatibility and the risk of accelerated biodegradation [51,52]. Surface treatments like anodizing and plasma spraying can also be used to modify the surface properties of Mg alloys. While anodizing improves the corrosion resistance by adding a protective oxide layer on the implant’s surface, plasma spraying creates a roughened surface and deposit a layer of biocompatible material on the implant’s surface to improve corrosion resistance and prevent accelerated degradation [53,54].

In addition, magnesium has a high ductility, which means that it can deform without fracturing, making it more resistant to mechanical failure. Being radiopaque, Mg can be easily visualized in imaging studies, such MRI and CT scans, although the resolution of X-ray can be relatively better for implants made of other high-density degradable metallic such as iron, zinc, and copper [6,55,56].

(iv) Bioactivity and Apatite Formation

**Figure 4:**
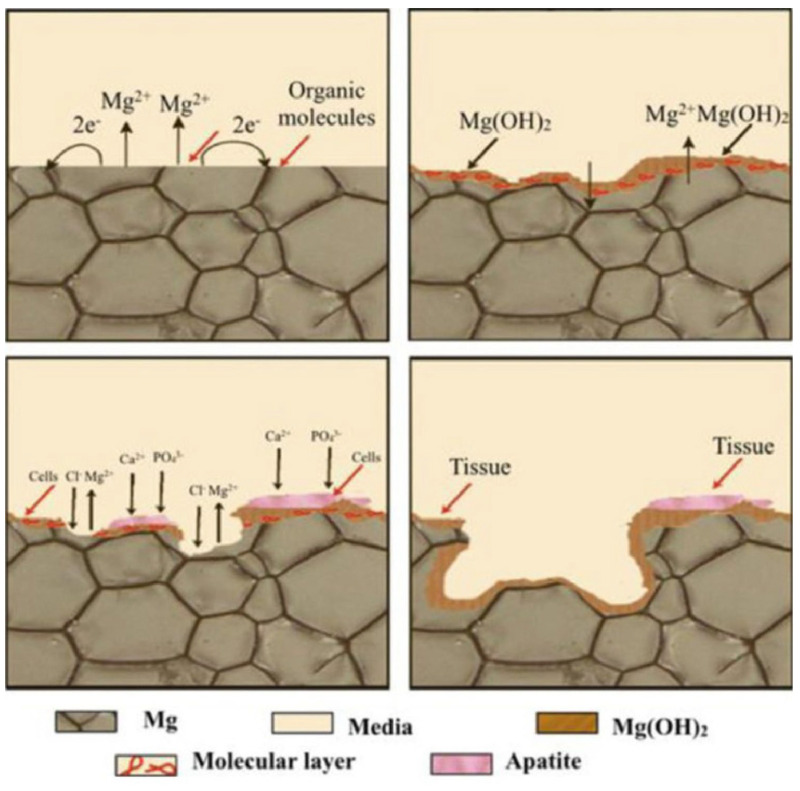
Degradation mechanism of Mg in vivo (Source: Tsakiris et al. [31], open access publication).

Bioactivity, which refers to the surface apatite layer, plays a major role in the osseointegration process. It refers to the generation of a layer of apatite made of inorganic compounds, such as calcium phosphates (CaP) and hydroxyapatite (HA), on the surface of implant due to the chemical reaction with calcium (Ca^2+^) and phosphate (PO_4_^3−^) ions present in the body fluids. Being essential components of bones and teeth, they facilitate the natural integration of implants with the neighboring tissues [6,31]. The mechanism of apatite formation involves a series of complex chemical reactions as explained below and as shown in Figure 4.

When Mg implant is placed in the body, the interaction between the surface of the implant and water produces magnesium hydroxide (Mg(OH)_2_) and hydrogen gas (H_2_). This reaction is given by:

$$\mathrm{Mg}+2H_{2}O\to \;\mathrm{Mg}{\left(\mathrm{OH}\right)}_{2}+{\;H}_{2}$$

The Mg(OH)_2_ then reacts with Ca^2+^ and PO_4_^3−^ ions present in the body fluids to form CaP and the same can be represented by:

$$\mathrm{Mg}{\left(\mathrm{OH}\right)}_{2}+{\;\mathrm{Ca}}^{2+}+{\;\mathrm{PO}}_{4}^{3-}\to {\mathrm{CaMg}}_{2}{\left({\mathrm{PO}}_{4}\right)}_{2}+2H_{2}O$$

The CaP layer then undergoes transformation into HA by incorporating additional Ca^2+^ and PO_4_^3−^ ions, as explained by the chemical reaction:

$${\mathrm{CaMg}}_{2}{\left({\mathrm{PO}}_{4}\right)}_{2}+4{\mathrm{Ca}}^{2+}+6{\mathrm{OH}}^{-}\to 5{\mathrm{Ca}}_{10}{\left({\mathrm{PO}}_{4}\right)}_{6}{\left(\mathrm{OH}\right)}_{2}+2{\mathrm{Mg}}^{2+}\;$$

The resulting HA layer on the surface of the Mg implant can act as a bridge between the implant and the surrounding bone tissue, promoting osseointegration and enhancing the biocompatibility of the implant. In general, the formation of the apatite layer is proportional to the bioactivity of biomaterial, and the same can be evaluated using XRD, SEM, and EDS analyses.

### In Vitro/In Vivo Studies and Clinical Trials on Mg-Based Implants

Several researchers have evaluated the performance and degradation of Mg-based temporary implants through in vitro and in vivo studies. The in vitro studies are primarily aimed at assessing the behavior of Mg-based implants in simulated physiological environment outside the living body. In these studies, the degradation behavior of Mg implants was assessed in terms of several factors, such as temperature, pH, and the presence of other ionic impurities, and the assessment of biocompatibility was carried out using systematic procedures like MTT assay, cell adhesion, cell proliferation, alkaline phosphatase activity (ALP), and blood compatibility [57]. Gray-Munro and Williams [58] investigated the biodegradation behavior of pure Mg in simulated body fluids (SBF) and found that the material corroded rapidly, with significant hydrogen evolution and a loss of mechanical strength within a few days of immersion. This study also reported on the formation of a surface layer made of magnesium hydroxide and magnesium oxide.

In another study, Wang et al. [59] reported that the corrosion resistance and biocompatibility of Mg–Ca alloy improves after coating with calcium phosphate. Further, it was also evident from the past research works that the addition of calcium, zinc, strontium, and rare earth alloying elements to Mg resulted in better cell viability and adhesion [60,61,62,63,64]. Similar improved cell viability and adhesion were also observed for HA and Ca_3_(PO_4_)_2_ coatings [59,65]. In vitro studies to understand the bioactivity of Mg implants have also been conducted and the findings confirmed the successful formation of surface apatite layer on most alloys [66,67]. In this regard, a few recent research works have highlighted the benefits of coatings like MgF_2_, Sr_3_(PO_4_)_2_, and fluoridated HA in improving the interfacial bioactivity of Mg implants [68,69,70]. Owing to their ability to resist bacterial invasion of the exposed hot tissue surface, Mg-based implants were also tested for antimicrobial activity. For example, Lock et al. [71] investigated the degradation behavior and antibacterial properties of Mg alloys in artificial urine for potential application as ureteral stent. Results from this study revealed that the ureteral stents made of pure Mg, Mg–Y, and AZ31 alloys significantly reduced the E. coli bacterial colony formation compared to polyurethane stents. In another study, Liu et al. [72] reported the good antibacterial properties of Mg–Al alloys against Staphylococcus aureus and Staphylococcus epidermidis bacteria. A similar antibacterial response was also reported for Mg alloys with copper, zinc, and rare earth [72,73,74]. The above-mentioned studies including several others concluded on the fact that the biocorrosion properties of Mg can be tailored by adjusting the alloy composition and protective coatings. 

Published articles have also investigated on the mechanical properties of Mg-based implant materials in simulated body fluids and similar environments. For example, Song et al. [75] found that the fatigue performance of AZ31B is remarkably inferior upon exposure to SBF than in air and they attributed it to the initiation and growth of corrosion pits, which acted as stress concentrators and promoted crack nucleation and propagation. In another study, Liu et al. [76] investigated the corrosion fatigue behavior of an extruded Mg–Zn–Y–Nd alloy in SBF and found that the fatigue strength of the alloy decreased with increasing number of cycles, although it exhibited good corrosion resistance with no significant corrosion pits or cracks on the surface after immersion in SBF. Hence, the authors attributed the decrease in fatigue strength to the formation and growth of microcracks initiated from pre-existing defects and corrosion pits. In this regard, Kashyzadeh et al. [77] published a critical review summarizing the effects of various alloying elements on the fatigue life and corrosion properties of Mg alloys. They concluded that the cyclic deformation behavior of Mg alloys is influenced by several factors, including alloying elements, microstructure, texture, loading conditions, and temperature. While the fatigue response in general is inferior compared to that of the traditional metallic biomaterials like titanium and stainless steel, Mg alloys exhibit sufficient fatigue resistance for biomedical applications, and the same can be further improved by several methods including alloying, surface modification, and grain refinement [78].

In this regard, Bowen et al. [79,80,81] compared the corrosion behavior of Mg and Fe wires and demonstrated that iron and Mg corrode differently in a physiological environment. While iron corroded locally, resulting in a large amount of voluminous corrosion product, Mg was found to corrode more uniformly, and its corrosion products comprised of Ca, Mg, P, and O. Additionally, the authors also proposed tensile testing as a quantitative methodology to assess the degradation of bioabsorbable materials. In general, the tensile strength and elongation at break exhibits a significant decline as the materials underwent degradation. For example, the complete corrosion can be conformed from “zero tensile strength”, while the fractional values are indicative of their respective corrosion rates. In this study [81], it was found that the time to achieve complete corrosion (i.e., zero tensile strength) was ~20–40 days for Mg and ~90–200 days for Fe, respectively. Hence, it can be deduced that the derived correlation provides a crucial framework for the prediction and evaluation of bioabsorbable magnesium stent performance.

Several in vivo studies have also been conducted on Mg-based implant materials in animal models and human subjects, with a primary aim of generating more realistic information about their degradation mechanism and biocompatibility under physiological conditions. Witte et al. [82] investigated the in vivo corrosion behavior of four different magnesium alloys (AZ31, AZ91, WE43, and ZK60) implanted in the femur of rats for up to 12 weeks. They found that the alloys exhibited significantly different degrees of corrosion, with ZK60 showing the highest corrosion rate and AZ31 showing the lowest alongside an increase in the pH of surrounding tissues due to the presence of corrosion products. However, the biocompatibility of all the alloys were reported as acceptable, and they were found to have assisted in the bone formation around the implants despite increased corrosion. 

Waksman et al. [83] examined the in vivo corrosion behavior of a Mg–Zr–Y–RE alloy stents in the coronary arteries of domestic or minipigs. The results were compared to that of stainless-steel stents coated with amorphous silicon carbide, which revealed the favorable biocompatibility of Mg alloy stents as they sustained arterial patency for up to 28 days post-implantation, without eliciting any significant inflammatory response. Based on these observations, the authors concluded that the investigated Mg alloy stents are safe and effective than the stainless-steel stents coated with amorphous silicon carbide. Another study [84] also reported an acceptable host response when the LAE442 Mg alloy was implanted in medial femur condyle of adult rabbits. In this study, the authors also commended the beneficial role of MgF_2_ coating that substantially reduced the corrosion rate of LAE442 alloy.

In a similar study, Wang et al. [85] evaluated the in vivo degradation of their patented Mg alloy (NZK, PRC Patent ZL 201010252357.0) containing Nd, Zn, and Zr in rabbit femur and found that the alloy degraded gradually over a period of 24 weeks without any significant adverse effects on the surrounding tissues. Hence, they concluded that their NZK alloy with adequate biocompatibility and mechanical strength is a potential orthopedic implant material. Sato et al. [86]) investigated the in vivo degradation behavior of AZ31 plates implanted into the tibia, head, back, abdominal cavity, and femur of male Wistar rats. In this study, the histological analysis of the tissues surrounding the plates revealed a normal wound-healing process with no statistically significant variation of histological scoring among the implantation sites at the end of 4 weeks. 

Sunil et al. [87] also reported no health abnormalities when AZ31 alloy was implanted into the femoral bone shaft of New Zealand white rabbits. In another study, the histological analyses following a 9-month implantation of ZEK100 alloy in the tibia of rabbits revealed generation of new bone tissues without any adverse effects [88]. Similar results were also reported by Makkar et al. [89] when Ca-added Mg alloys were implanted into rabbit femur. In this regard, several other research works have also demonstrated the benefits of alloying elements like zinc, calcium, and rare earth elements in improving the in vivo corrosion response of Mg alloy implants [90,91,92,93].

Mao et al. [94,95] developed new Mg alloys (Mg–2.5Nd–0.2Zn–0.4Zr and Mg–2.2Nd–0.1Zn–0.4Zr, denoted as JDBM and JDBM-2, respectively) with extremely homogenous degradation behavior compared to AZ31. The in vitro results revealed no significant adverse effect on the cell viability and growth when tested using human vascular endothelial cells. Similarly, the in vivo assessment confirmed long-term stability and structural integrity in blood vessel for up to 6 months. However, the alloys also exhibited compromised foreign body response as determined by human peripheral blood-derived macrophage adhesion, foreign body giant cell (FBGC) formation, and inflammatory cytokine and chemokine secretion. In a related study, Liu et al. [96] investigated a simple one-step process to introduce a cross-linked 3-amino propyltrimethoxysilane (APTES) silane physical barrier layer on the surface of PLGA electrostatic-sprayed Mg–Zn–Y–Nd alloys. The results of nanoscratch and electrochemical tests revealed superior adhesion strength and in vitro anticorrosion behavior due to the pre-treatment of PLGA with APTES. Cell morphology and proliferation data demonstrated compatibility for both umbilical vein endothelial and vascular smooth muscle cells. The animal study was conducted by implanting this alloy into porcine coronary arteries and the results revealed benign tissue compatibility as well as re-endothelialization without thrombogenesis or in-stent restenosis at the 6-month follow-up.

While Mg implants are generally tested for in vitro degradation in accordance with ISO10993, it is worth noting that the set guidelines do not clearly define the extract preparation procedure for biodegradable materials like Mg. Hence, the correlation of in vivo and in vitro degradation requires a systematic approach. This is especially true in the case of Mg as the Mg alloy extracts are known to exhibit false cytotoxic behavior due to high pH and osmotic shock. For example, the faster surface degradation of nano-HA-coated Mg alloy will often lead to the falsification of results when tested for cell adhesion even though the alloy exhibits a better cell adhesion to bone marrow stem cells [97,98,99]. 

Over the past decade, several clinical trials have been conducted to evaluate the safety and efficacy of Mg implants. Table 4 lists their key findings as available from the open literature.

## 4. Applications of Magnesium-Based Biodegradable Temporary Implants

Magnesium-based temporary implants have emerged as a promising alternative to traditional metallic implants due to their biocompatibility, biodegradability, and acceptable mechanical properties. Their ability to degrade over time and be safely absorbed by the body reduces the need for removal surgery and the potential for long-term complications. For these reasons, they are used as implants in orthopedics, dentistry, cardiology, and several other medical fields. 

In orthopedic surgeries, implants are used to stabilize and support the bones or joints that have been damaged or weakened by injury or disease. As Mg implants promote bone formation and tissue regeneration and have a lower rate of infection than the traditional metallic implants, they are an attractive option for orthopedic surgery [40]. For example, Mg plates and screws are extensively used to repair bone fractures. Bone fracture fixation is one of the most common applications of temporary implants, and the current gold standard is the use of metal plates and screws. Since biocompatible metals like titanium and stainless-steel pose several limitations like stress shielding, implant failure, and the need for a second surgery for removal, Mg-based temporary implants with comparable properties as cortical bone started receiving stupendous attention in recent years, and they are being used in the fixation of various fractures such as femoral, tibial, and mandibular fractures [91]. Mg-based temporary implants have also emerged as a promising alternative to traditional metal implants in spinal fusion surgery. Spinal fusion is a surgical procedure that involves the fusion of two or more vertebrae to stabilize the spine. Currently, it relies on titanium and stainless-steel implants associated with the above-mentioned limitations and challenges [125]. In this regard, recent studies highlight the potential of Mg-based temporary implants for spinal fusion surgery [126,127]. Similarly, Mg-based temporary implants have also shown potential in maxillofacial surgery that involves the treatment of injuries and defects in the face, jaw, and neck. In a study by [128], Mg-based implants were demonstrated to be effective in maxillofacial surgery, suggesting that they have potential as an alternative to traditional metal implants. Mg alloys have also been tested as a potential material for hip and knee replacements [129].

Cardiovascular disease is one of the leading causes of human death worldwide, and the use of stents is a common treatment option for patients with narrowed or blocked arteries. Since, traditional metallic stents can cause complications, such as restenosis and thrombosis, Mg-based stents are being investigated as they exhibit a lower risk of restenosis [130]. Mg alloys are also being explored in the making of temporary scaffolds for endovascular treatment of aneurysms, where they gradually degrade and are replaced by new tissue [131]. Dental is another interesting area where Mg implants can be used to treat patients with missing or damaged teeth. As traditional metallic implants can cause complications such as peri-implantitis, a condition that leads to bone loss and implant failure, Mg-based implants are being explored for dental applications [132]. Other potential applications include neurosurgery, ophthalmology, and urology. For example, Mg-based plates are being tested as a potential treatment option for cranial fractures. Similarly, Mg-based stents have been explored in the treatment of ureteral strictures [71,133,134,135].

## 5. Commercially Available Magnesium-Based Temporary Implants

Over the past decade, several companies have developed magnesium implants for various medical applications. They are focused on the development of novel Mg alloys that can be designed to degrade over time and be replaced by natural tissue or bone. Currently, most of them use Mg–Y–Zn alloys as they have a degradation rate that can be tailored by adjusting the alloy composition. Table 5 lists a few commercial implants approved by the regulatory bodies, such as US Food and Drug Administration (FDA) and the European Medicines Agency (EMA).

## 6. Fabrication Methods Applicable for Mg-Based Temporary Implants

**Figure 5:**
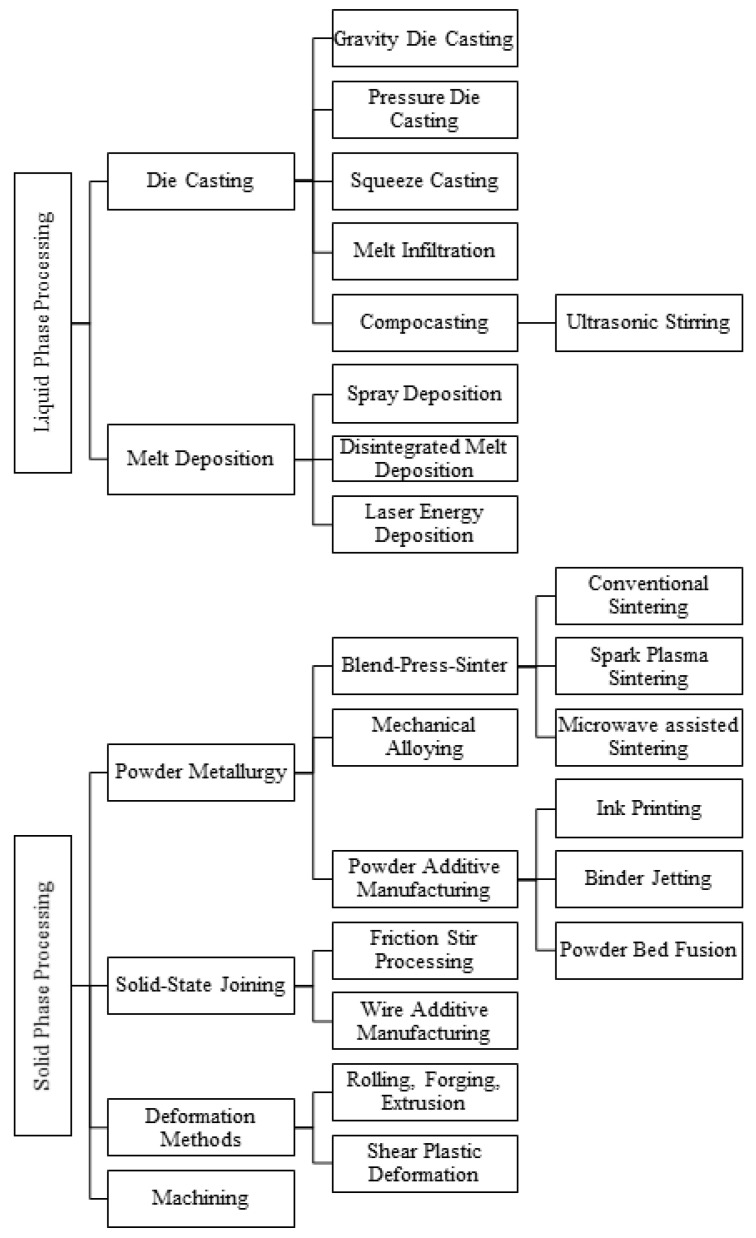
Classification of fabrication methods applicable to Mg-based materials.

A variety of methods can be employed for the shaping of Mg-based temporary implants, and they can be broadly grouped under either liquid or solid-state processing methods as shown in Figure 5. Liquid-state processes involve metal casting and shaping methods, such as melt infiltration, stir casting, and melt deposition. Similarly, the solid-state processing involves powder metallurgy, solid-state joining, plastic deformation, and machining methods. Each of these methods are discussed briefly in the following section.

(a) Die Casting

**Figure 6:**
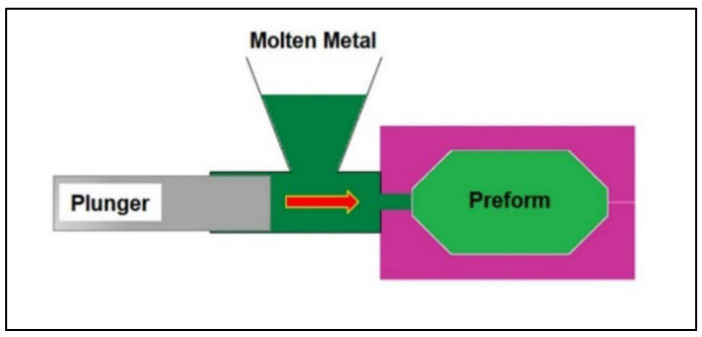
Schematic of pressure die casting.

This process involves the injection of molten Mg into a die or mold cavity to generate the required shape. It is typically carried out in three steps: (i) preparation of die, during which the die or mold cavity is coated with a lubricant to prevent molten Mg from sticking to its surface; (ii) melting, followed by the injection of molten Mg; and (iii) ejection of Mg casting upon solidification (Figure 6).

**Figure 7:**
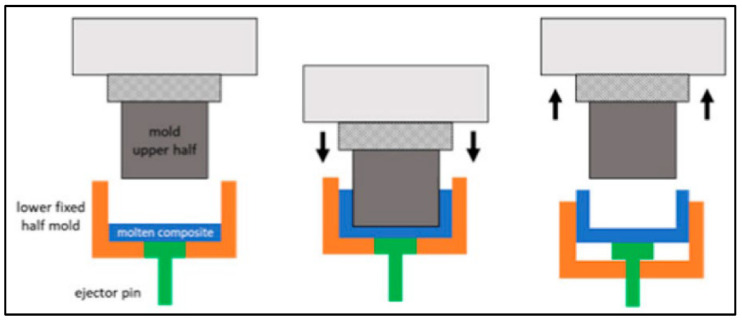
Schematic of squeeze casting process (Source: Seetharaman et al. [136], open access publication).

Squeeze Casting: This method involves application of pressure during the solidification of molten Mg, which helps to create a refined microstructure and eliminate residual porosity or voids in the casting (Figure 7). The preheated mold is first filled with molten Mg, followed by the application of pressure on to the mold while the melt is still in the semi-solid state. The high pressure applied during solidification helps to generate a final product with fine and homogeneous microstructure resulting in superior strength and ductility.

**Figure 8:**
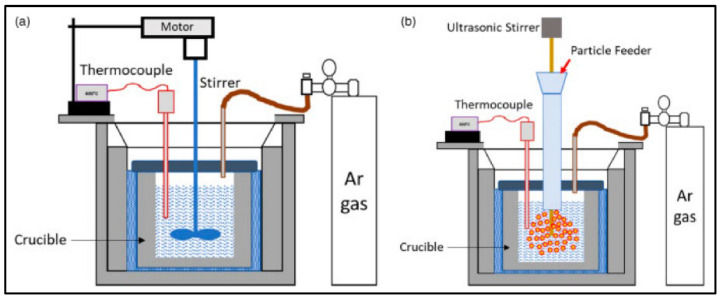
Schematic of (**a**) mechanical stirring and (**b**) ultrasonic stirring-assisted compocasting (Source: Seetharaman et al. [136], open access publication).

Compocasting: This method applies to composite fabrication as it facilitates the addition of reinforcing phases, such as ceramic fibers or particles to molten Mg. As reinforcements tend to agglomerate, careful attention must be paid to ensure their effective dispersion in Mg matrix, which can be achieved using a mechanical or ultrasonic stirrer (Figure 8). The resulting composite slurry can be solidified following either gravity or pressure die casting. In some cases, wetting agents are also applied on to the reinforcement to improve the interfacial bonding and to avoid any unwanted reaction products [136]. Mg composites produced by this method generally exhibits improved strength, wear resistance, and thermal stability compared to that of Mg and Mg alloys.

(b) Melt Deposition

In general, deposition methods including plasma spray deposition, disintegrated melt deposition (DMD), and laser-aided directed energy deposition and have been successfully used to fabricate Mg alloys and composites.

**Figure 9:**
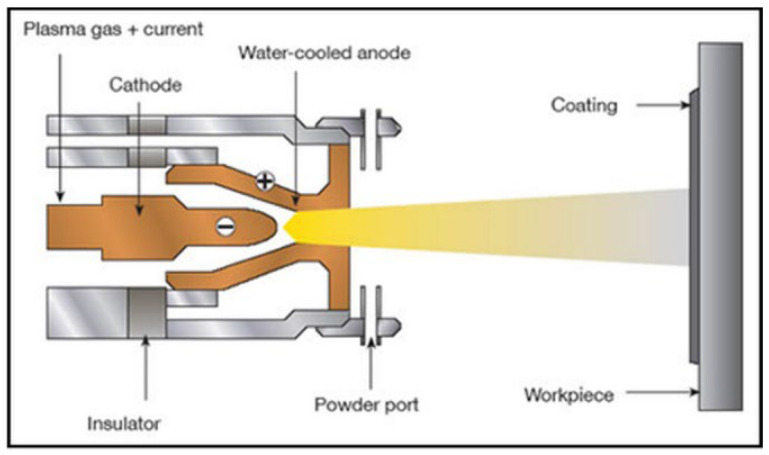
Plasma spray deposition (Source: R. Heimann [137], open access publication).

Plasma spray deposition: This involves the use of a plasma jet to melt and deposit a Mg powder feedstock onto a substrate surface (Figure 9). The process begins by feeding a Mg powder feedstock material into a plasma torch, where it is rapidly melted and propelled onto the substrate surface. The deposited Mg melt upon cooling and solidification forms a thin film on the substrate. Although this method widely used for the application of coatings, it can be optimized to fabricate bulk materials and complex geometries. This method can also be applied to produce Mg composites, wherein the particle reinforcements can be directly injected into the spray of molten metal before being deposited onto a substrate, and the properties of deposition can be controlled by adjusting the feedstock characteristics and processing parameters, such as the plasma gas flow rate, temperature, and spray distance.

**Figure 10:**
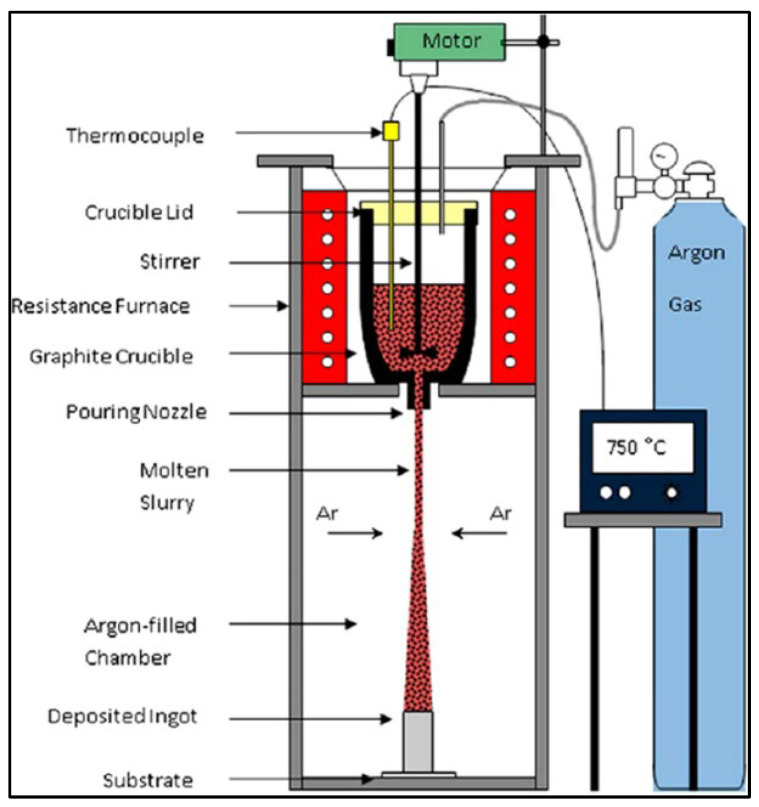
Disintegrated melt deposition (Source: Malaki et al. [138], open access publication).

Disintegrated melt deposition: It combines the advantages of cost-effective compocasting and spray processing methods as it involves the vortex mixing of molten Mg and its deposition onto a metallic substrate after disintegration by jets of inert gases (Figure 10). Unlike spray deposition, this liquid-state method is highly suitable for developing bulk materials with a fine grain structure as it employs a lower impinging velocity, and the recovery of the poured material is almost 100% with no overspray powders [138].

**Figure 11:**
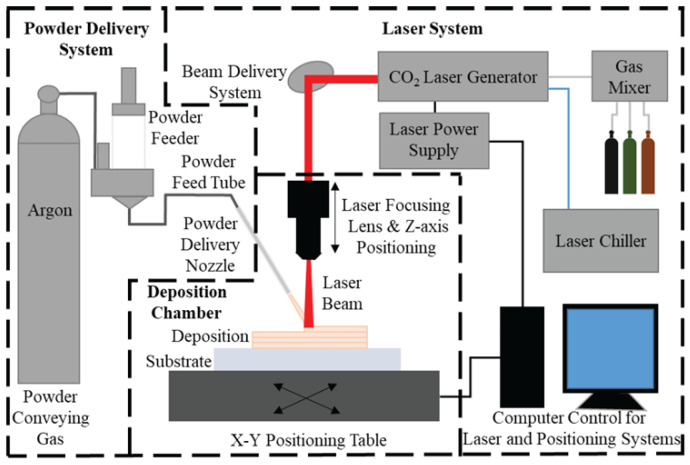
Setup for laser direct deposition as proposed by Pappas and Dong [139] (SFF symposium proceedings, open access publication).

Laser energy deposition: This process involves a carrier gas to deliver the Mg powder feedstock, which upon interaction with the laser energy source gets melted and then deposited onto a substrate (Figure 11) [139]. The substrate is moved in such a way that the process of melting and deposition continues layer by layer to generate a three-dimensional object.

(c) Powder Metallurgy Methods

**Figure 12:**
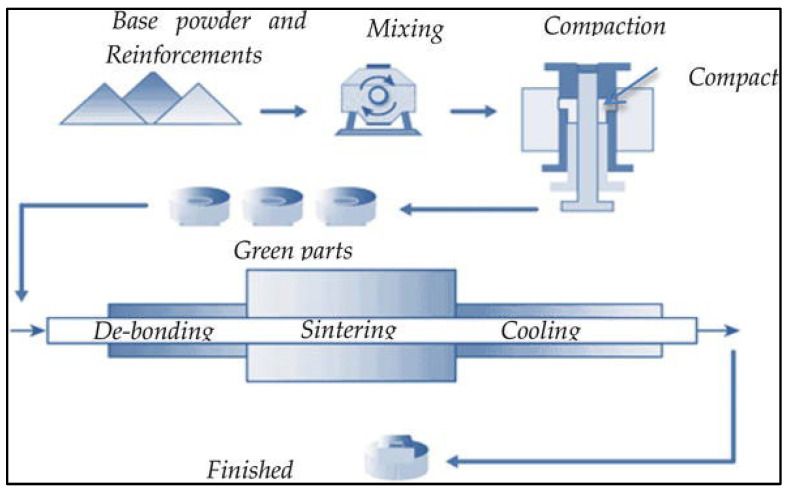
Schematic showing conventional powder metallurgy method involving mixing of raw materials, compaction, and sintering of green parts (Source: HG Prashantha Kumar and M Anthony Xavior, [141], open access publication).

Simple blending of powder particles: In this method, a powder blend prepared by the simple mixing of raw material feedstock is hot or cold compressed into a billet of required dimensions. The billet is then canned, degassed, and sintered at temperature closer to the solidus temperature of the matrix alloy (Figure 12). The sintering process involves heating the green compact billets prepared by simple powder blending and mechanical alloying methods to a temperature closer to the solidus line of the matrix alloy. Here, the atomic diffusion facilitates the formation of inter-particle bonds between the powder particles [140]. In most cases, the sintering of green powder compact also facilitates the microstructural recrystallization for strengthening alongside densification and removal of residual lubricant. It is also important to note that this method is not effective for producing fiber-reinforced Mg composites as fibers often get damaged under the high pressure during pressing [136,138].

**Figure 13:**
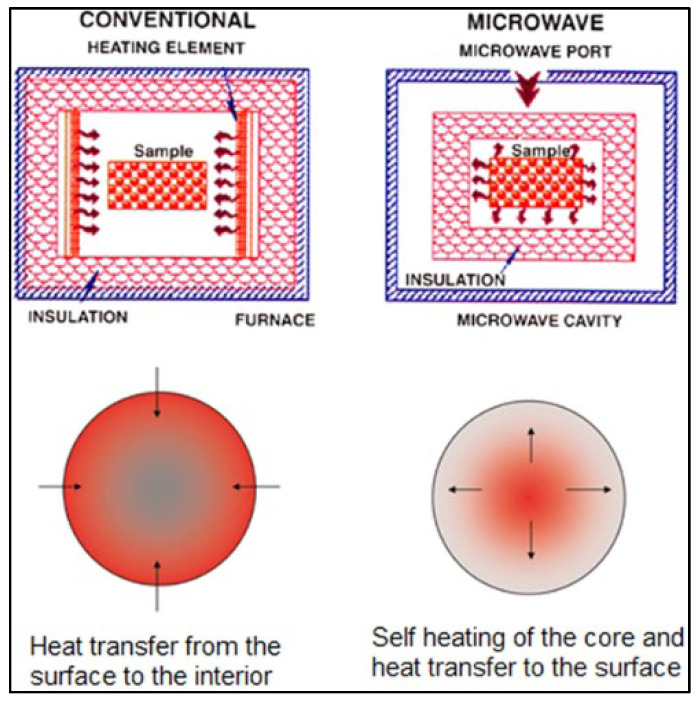
Schematic of microwave sintering and heat transfer principle (Source: Matli et al. [142], open access publication).

In this regard, microwave sintering has recently emerged as an energy-efficient technique to consolidate metal powders. It involves the self-heating of the material core due to dielectric and magnetic losses resulting from the interaction between the electric and magnetic fields, and the subsequent transfer of heat from the core to the surface of the material [142]. As microwaves exhibit an inverse temperature distribution, the microwave heating is generally rapid, thereby reducing the processing time by >80%. This is unlike conventional heating, where the transfer of heat by conduction, convection, and radiation occurs from the surface to the interior of the material, and hence is relatively more time-consuming (Figure 13). While microwave processing has been largely limited to ceramics in the past, there are several recent papers confirming the feasibility to process metallic materials.

Mechanical alloying: This method follows similar steps except for the fact that the powder raw materials are subjected to prior treatment by ball milling, which involves the repeated cold welding, fracturing, and re-welding of powder particles that results in their local melting and consolidation due to the frictional heat developed at the particle interface [143,144]. The mechanically alloyed powders are then densified by either cold- or hot-pressing techniques as explained earlier. This method can be generally applied to develop a range of equilibrium/non-equilibrium alloys and composites as it ensures the homogenous distribution of reinforcing constituents and the generation of a high volume of dislocation densities.

**Figure 14:**
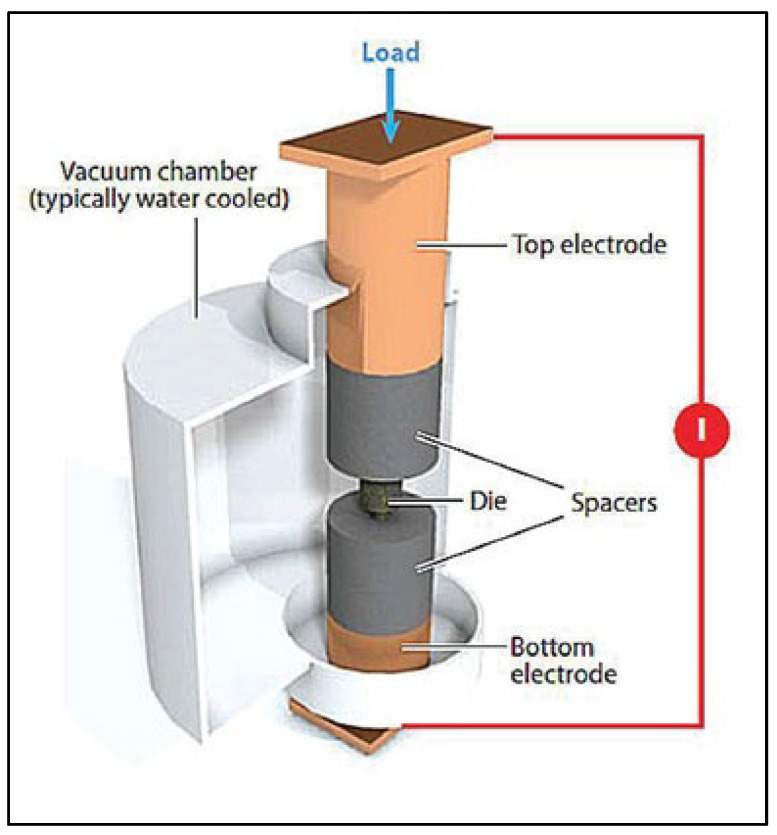
Schematic of spark plasma sintering (Source: Franceschin et al. [146], open access publication).

Spark plasma sintering: This process utilizes a uniaxial force and a pulsed (on/off) direct electrical current (DC) to consolidate the powder raw materials of Mg (Figure 14), and it involves three major stages: (i) plasma heating, (ii) joule heating, and (iii) plastic deformation [145]. During plasma heating, a localized and momentary heating of particle surfaces occurs due to the electrical discharge between powder particles. The flow of DC current between the particles then results in necking due to the Joule heating effect, which increases the diffusion of atoms at the particle interface. The heated material becomes soft in the final stage and deforms under the application of uniaxial force. Therefore, the spark plasma sintering (SPS) technique combines the benefits of atomic diffusion and plastic deformation to achieve densification of powder compact by up to 90%. It is also important to note that the SPS process is usually carried out at a low atmospheric pressure to ensure rapid consolidation.

Powder additive manufacturing: It is a category of additive manufacturing processes that involves fusing of powder material layer by layer to create a three-dimensional object. It includes processes such as direct ink writing, ink jetting, binder jetting, powder bed fusion, and directed energy deposition methods.

**Figure 15:**
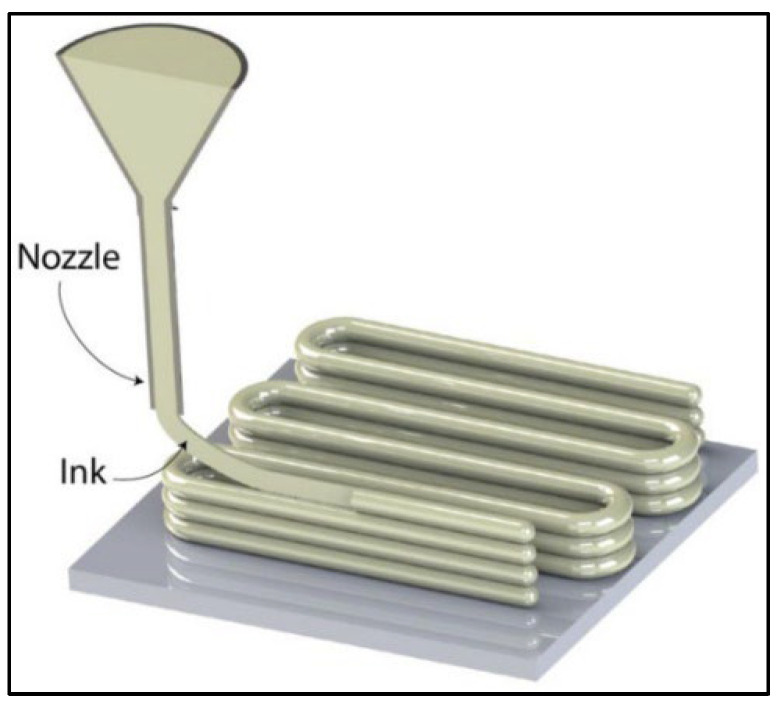
Schematic of a direct ink writing 3D printing process (Source: A. Kantaros [147], open access publication).

Direct ink writing (DIW): This process involves the extrusion of magnesium powder together with a binder material through a nozzle, layer by layer, to create a solid object (Figure 15). The printed object is then dried, cured, and sintered at high temperatures to fuse the magnesium particles together. DIW is versatile and can produce complex structures with tailored properties, making it a promising option for developing personalized medical implants [147].

**Figure 16:**
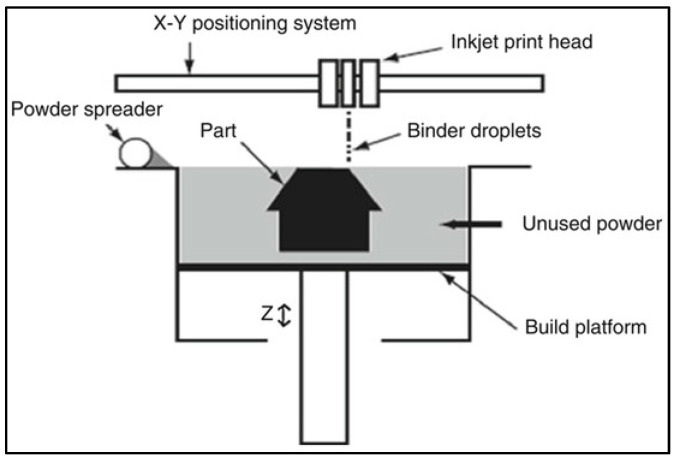
Schematic of binder jetting printing process (Source: S. Mirzababaei and S. Pasebani [148], open access publication).

Binder jetting: In this process, a layer of Mg powder is first deposited onto a substrate, followed by the selective application of binder material to join the powder particles together (Figure 16) [148]. This process is repeated layer by layer until the desired object is formed. The resulting “green” part is then subjected to a sintering process, where the binder is removed and the magnesium powder particles fuse together, creating a solid metal part. Metal binder jetting offers high precision and can produce complex geometries, making it a promising method for the production of magnesium implants with tailored properties.

Ink Jetting: It involves the use of an inkjet printhead to selectively deposit the solution containing Mg ions onto a substrate in a layer-by-layer manner. The deposited solution is then dried and cured, and the process is repeated until the desired object is formed. The object is then sintered to fuse the magnesium particles together and create a solid structure. Ink jetting is a promising method for the fabrication of magnesium implants due to its ability to create complex structures with high precision and accuracy, making it a potential option for developing customized implants.

**Figure 17:**
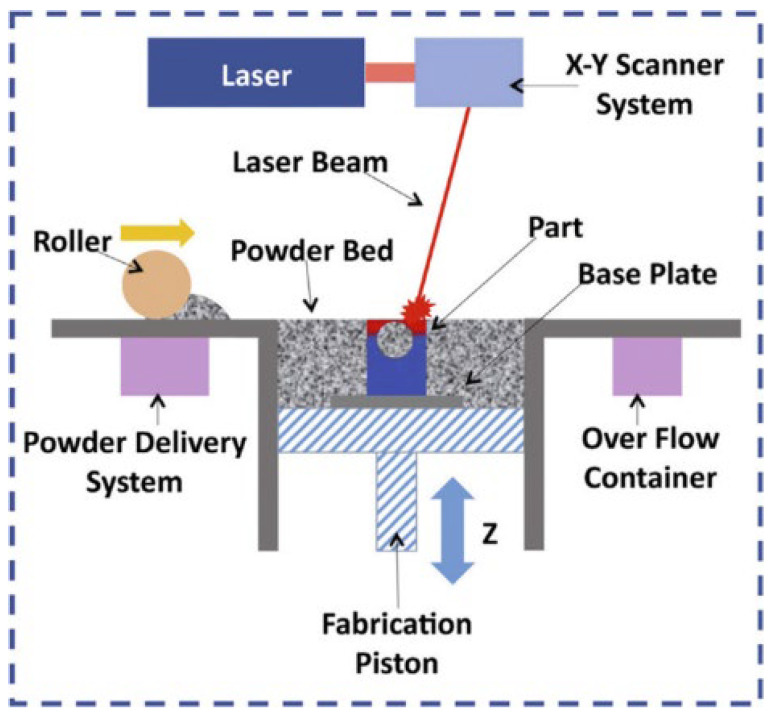
Schematic of LPBF process for Mg processing as reported by Kurzynowsk, et al. [149] (open access publication).

Laser powder bed fusion: It involves spreading a layer of magnesium powder onto a build platform, followed by the selective melting of the powder using a laser or electron beam (Figure 17). This process is repeated layer by layer until the desired object is formed. The melted magnesium solidifies almost instantly, creating a solid metal part. The resulting part is then subjected to a post-processing step, such as polishing or heat treatment, to improve its properties. Powder bed fusion can produce highly complex structures with high accuracy and precision, making it a promising method for producing customized magnesium implants with specific geometries and properties.

(d) Solid-State Joining Methods

**Figure 18:**
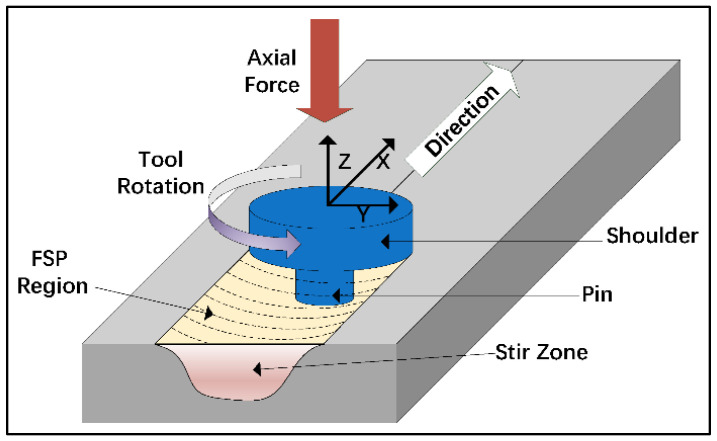
Schematic of friction stir processing (Source: Li et al. [150], open access publication).

Friction stir processing: It is a solid-state welding method used to fabricate Mg-based materials through surface modification [150]. In this process, the material undergoes severe plastic deformation, as shown in Figure 18, which results in a homogeneous fine-grained microstructure. Being a solid-state method, FSP does not involve the melting of materials, and it effectively avoids defects like porosities and hot cracks that are commonly observed during the solidification of molten composite slurry. For composites, the effective dispersion of reinforcements depends on the frictional heating at the interface between a rotating tool and the matrix material.

**Figure 19:**
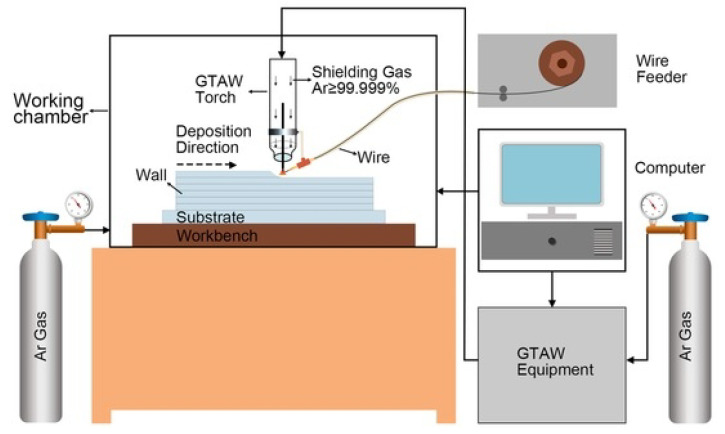
Schematic of WAAM set up used by Guo et al. [151], (open access publication).

Wire additive manufacturing (WAAM): It involves feeding a Mg wire feedstock through a nozzle and melting it using a heat source like an arc welding torch, laser, or electron beam (Figure 19). In general, the wire is typically fed through the nozzle or the powder feeder at a constant rate, while the heat source is moved across the substrate or the previous layer according to a pre-programmed pattern. This allows for the creation of a controlled molten pool, which fuses the wire to the substrate or the previous layer as the heat source moves along the pattern. The process is repeated layer by layer until the desired part is completed.

(e) Deformation Processing Methods Applicable to Magnesium Alloys

Magnesium alloys can be shaped and formed into different shapes using deformation methods such as extrusion, forging, rolling, and drawing. These methods involve the application of excessive force in a controlled manner to induce plastic deformation, resulting in a change of shape. In extrusion process, a heated billet of magnesium alloy will be forced through a die to produce a long continuous shape of uniform cross-section, which can then be cut into desired lengths and further processed as needed. Similarly, in forging, the heated billet will be subjected to compressive force using a hammer or a press in either one or more directions. Rolling on the other hand involves passing the billet through a set of rollers to reduce its thickness and change its shape. Due to the deformation-induced grain refinement, all these processes are capable of producing wrought alloy cross-sections with improved strength and ductility. Drawing is a similar process where the alloy wire or tube will be pulled through a die to reduce its diameter and change its shape. In this context, several severe plastic deformation (SPD) methods, such as Equal Channel Angular Pressing (ECAP), High-Pressure Torsion (HPT), and Accumulative Roll Bonding (ARB), have also been developed and applied to Mg alloys. These methods induce severe plastic strain (von Mises strain in excess of 2) the material to generate sub-micro/nano-crystalline grain architecture, leading to superior mechanical properties [152,153].

(f) Machining of Magnesium

Mg alloys are known for their excellent machinability due to their low cutting forces, low power requirements, and high specific cutting energy. In general, the machining process involves removal of excess material from a workpiece using cutting tools and it can be classified into two main categories: conventional machining and non-conventional machining [154,155,156]. Conventional machining methods, such as turning, milling, drilling, and tapping, are widely used for Mg alloys, and they involve cutting tools made of high-speed steel or cemented carbide. The machining parameters, such as cutting speed, feed rate, and depth of cut, are usually optimized for specific alloy composition to achieve maximum material removal while minimizing the tool wear and surface roughness.

In addition to the benefits of excellent machinability, machining of Mg also comes with certain limitations, the most notable being its self-ignition characteristics. Being highly flammable, Mg can self-ignite upon exposure to raised temperature or sparks generated during the machining process. This presents a significant safety concern for machinists and can lead to fires or explosions if not properly managed. Therefore, special precautions must be taken to mitigate this risk, which includes the use of specifically designed cutting fluids as well as the implementation of fire suppression systems and other safety measures. Additionally, machining parameters, such as cutting speed, feed rate, and depth of cut, must be carefully controlled to prevent the generation of excessive heat that can ignite the material [154,157].

In this regard, recent advancements include the implementation of cryogenic cooling and minimum quantity lubrication techniques to improve machining performance and reduce the risk of fire hazards associated with the use of conventional cutting fluids [158]. Further, the use of new cutting tool materials and coatings has also been found to be beneficial. For example, diamond-coated tools and cubic boron nitride (CBN) tools have shown to have high wear resistance, improved surface finish, and reduced risks associated with self-ignition and flammability [159,160].

The benefits and limitations of the above-mentioned processing benefits are outlined in Table 6.

## 7. Current Challenges and Recommendations

There are several challenges associated with Mg-based biodegradable temporary implants and some of them are listed below:

- Rapid corrosion: It is the major challenge associated with Mg-based biodegradable implants. Being highly reactive, Mg corrodes rapidly in the presence of bodily fluids, making it difficult to control the degradation rate of the implant. Balancing the corrosion rate is crucial, as a high rate could lead to premature failure.
- Complex geometry of implants: The degradation behavior of Mg-based implants varies depending on the implant’s surface area-to-volume ratio and location in the body. Although controlling these variables can be challenging, it is necessary to ensure that the implant degrades in a controlled and safe manner.
- Inflammation due to corrosion products: While Mg is biocompatible, its degradation products can cause inflammation and tissue damage. Thus, developing better biocompatible magnesium-based materials is crucial to avoid negative side effects.
- Another key challenge associated with Mg implants is its susceptibility to stress corrosion cracking (SCC). In general, Mg implants can experience SCC when subjected to stress in a corrosive environment, leading to localized corrosion and cracking, resulting in premature failure. As the mechanism of SCC and its severity heavily depends on the alloy microstructure, incorporation of alloying elements (e.g., Al, Mn, and RE) and the control of grain size and texture are found to be beneficial in improving the resistance to SCC. Similarly, the application of protective coatings and surface treatments also act as barriers against corrosion to delay or prevent SCC. In this regard, the design of implants can also be optimized to reduce stress concentration and applied stress to reduce the risk of SCC.

As addressing the above challenges is critical for the successful development and implementation of Mg-based biodegradable implants, the following recommendations are proposed:

- Enhance mechanical properties: Mg-based implants must have adequate mechanical properties to provide sufficient support and stability during the implantation period. Hence, novel Mg alloys with improved mechanical properties are required for the development of high-performance Mg-based implants.
- Improve corrosion resistance: Since Mg-based implants exhibit a faster degradation rate in body fluids, coatings are recommended to control the corrosion rate and hence improve the corrosion resistance of Mg-based implants.
- Optimize biodegradation rate: As biodegradation is an essential factor for the successful application of Mg-based implants, the use of alloying elements, grain size refinement, and surface treatments must be explored to control the biodegradation rate of Mg-based implants.
- Develop appropriate manufacturing techniques: Appropriate manufacturing techniques are essential for the development of Mg-based implants with controlled microstructure and mechanical properties. Researchers should explore different manufacturing techniques, such as additive manufacturing and powder metallurgy, to optimize the microstructure and mechanical properties of Mg-based implants.

In addition, it is also important to build confidence on Mg-based implants by implementing the following.

- Conducting long-term clinical studies: Long-term studies should be conducted to evaluate the biodegradation rate, biocompatibility, and mechanical stability of Mg-based implants over extended periods. This will help to boost the confidence on Mg-based temporary implants over permanent implants.
- Standardizing test protocols: The testing protocols for Mg implants must be standardized to assess key aspects such as biodegradation rate, biocompatibility, and mechanical properties.

## Figures and Tables

**Table 1:** Characteristic differences between permanent and temporary implants.

Characteristics	Permanent Implants	Temporary Implants
Intended use	Designed to replace or enhance a body part or function that has been lost or compromised, such as a hip replacement, dental implant, or pacemaker.	Designed for short-term applications such as stabilizing bone fractures, providing support during tissue healing, or delivering medication to a specific site in the body.
Examples	Hip replacements, dental implants, pacemakers, breast implants, and joint replacements.	Sutures, splints, stents, temporary dental crowns, drug delivery systems.
Duration of placement	Designed to remain in the body for an extended period of time, often for the patient’s lifetime.	Intended to be removed after a certain period of time, ranging from a few days to several years.
Design considerations	Requires more complex design considerations to ensure their longevity, durability, and compatibility with the body. They are typically made from materials that are biocompatible and can withstand the stresses and strains of daily use, such as titanium, stainless steel, or ceramic.	Design and choice of material depends on the application. Can be made using materials that can be absorbed by the body or easily removed. For example, sutures are made from absorbable materials that break down over time and are absorbed by the body’s tissues. Splints and casts are made from materials such as plaster or fiberglass that can be easily removed once the injury has healed.
Surgical procedure	Requires invasive surgical procedures, such as the insertion of a hip replacement or the placement of a dental implant.	Temporary implants require multiple invasive surgical procedures. However, the rectification surgery for implant removal can be avoided if biodegradable materials are employed as temporary implants
Biocompatibility and corrosion	The material should be non-toxic and not cause an immune response or rejection by the body. The material should not corrode or degrade in the body’s harsh environment.	The material should be non-toxic and not cause an immune response or rejection by the body. Temporary implant must be designed in such a way that it either remains inert (for removable implants) or degrade over time in a controlled fashion (in the case of biodegradable implants).
Mechanical Properties	The material should be able to withstand the body’s natural wear and tear for a long period of time without degrading or breaking down. They should have high strength, stiffness, and durability for long-term support.	Sufficient strength to provide temporary support. The material should be flexible and able to conform to the shape of the body part where it is inserted.
Materials	Metallic implants, such as titanium, cobalt-chrome, and stainless-steel alloys, used in joint replacements, dental implants, and cardiovascular devices. Ceramic implants, such as alumina and zirconia, used in hip and knee replacements, dental implants, and spinal implants. Polymer implants, such as polyethylene, PEEK, and UHMWPE, used in joint replacements, spinal implants, and cardiovascular devices. Composite implants, such as carbon-fiber-reinforced polymers, used in orthopedic and sports medicine applications.	Biodegradable metallic implants, such as magnesium and iron, used in orthopedic and cardiovascular applications. Biodegradable polymer implants, such as polylactic acid (PLA) and polyglycolic acid (PGA), used in bone fixation, drug delivery, and tissue engineering. Resorbable ceramics, such as tricalcium phosphate (TCP) and hydroxyapatite (HAP), used in bone grafts and tissue engineering. Natural materials, such as collagen and fibrin, used in wound healing, tissue engineering, and drug delivery.

**Table 2:** Properties of implant materials (alloys and composites) [34,44].

Alloy	Tensile Strength (MPa)	Yield Strength (MPa)	Modulus of Elasticity (GPa)	Elongation (%)	Corrosion Rate (mm/year)
AE42	230	145	45–55	6	-
AE44	245	142	52–57	10	−0.2
AM100A	150–275	83–110	45–60	2–4	-
AM20	210	90	-	20	-
AM50	230	125	45	8	-
AM60	220	130	45–50	6	-
AZ31	240–290	150–220	45	9–21	0.1–0.3
AZ61	195–310	180–240	44–50	12–16	0.1–0.3
AZ63	200–275	97–130	45	6–12	-
AZ80	315–380	215–275	45	5–11	-
AZ81	275	85	45–65	15	-
AZ91	165–275	80–195	44–47	2.3–4.5	0.1–0.3
EQ21	234	172	-	2	-
EQ22	275	205	-	4	-
EZ33	160	105	-	3	-
WE43	235	190	45–55	4–10	0.05–0.1
WE54	270	190	45–60	4	-
ZE41	205	140	50	3.5	-
ZE63	295	190	-	7	-
ZEK100	250–280	140–200	45–50	10–15	0.02–0.05
ZK51	276	165	-	3	-
ZK60	305–365	200–305	-	11–16	-
MgCa0.4	190–230	72–166	-	21–34	-
MgCa0.8	145–185	70–100	45–55	10–20	0.2–0.3
MgCa	165–315	81–230	-	1.6–19	0.02–0.31
MgSn_0.25–3_Ca_0.2–1.5_	240	-	-	8–9	-
MgSr_0.5_	98	44	-	4.0	-
MgSr_0.3_Ca_0.3_	107	52	-	8.8	-
MgZn_1_Mn_1_Sr_0.25–1_	255–280	206–241	-	7–18	0.5–2.0
MgSrY	75–115	45–71	-	5–8	0.7–12
MgZn6	280	169	42.3	18.8	2.32
MgZn_1_Ca_0.5_	210	-	-	44	3.91
MgZn_4_Ca_0.2_	185–297	60–240	-	12.5–21.3	1.98–2.05
MgZn_4_RE_0.5_	142	110	-	8.9	0.105
ZX21	190–240	150–200	40–50	5–15	0.05–0.1
ZQ63	170–320	100–300	42–45	4–14	0.005–0.02
QX120	170–240	125–320	45–50	10–24	0.005–0.008
Mg/(10–30)Al_2_O_3_	200–240	160–180	50–60	5–10	0.005–0.03
Mg/(10–30)ZrO_2_	190–230	140–170	40–60	5–10	0.01–0.05
Mg/(5–20)TiO_2_	190–220	130–150	20–30	5–10	0.001–0.02
Mg/(10–30)HA	160–220	130–200	40–60	8–15	0.01–0.1
Mg/(10–30)TCP	170–220	100–150	40–50	8–12	0.02–0.08
Mg/(5–20)PLA	160–200	120–150	4–6	5–10	0.005–0.02
Mg/(5–20)PGA	200–240	170–200	5–6	3–6	0.001–0.01
Traditional Implant Materials					
Ti6Al4V	880–900	790–800	114	10–20	negligible
CoCrMo	1050–1300	800–1000	230	20–30	negligible
Stainless steel (316L)	520–700	190–260	200	40–50	negligible
Polyetheretherketone (PEEK)	90–120	70–100	3–4	50–100	negligible
Polylactic acid (PLA)	50–70	20–50	3–4	5–10	negligible
Polyglycolic acid (PGA)	30–50	10–30	1.5–2.5	10–20	negligible

**Table 3:** Effects of different alloying elements on the biocompatibility of magnesium.

**Alloying Element**	**Biocompatibility**	**Presence in Human Body**	**Presence in Blood Serum**
Zinc	Essential micronutrient and biocompatible. Acts as antimicrobial agent and prevents the growth of bacteria on the implant surface. Promotes osteogenesis and angiogenesis.	2 g	46 μmol/L
Calcium	Plays a crucial role in bone formation. The release of Ca^2+^ ions can stimulate cell growth and differentiation.	1100 g	0.919–0.993 mg/L
Rare earth elements (REEs)	REEs in general (for example, like cerium, neodymium, and yttrium) modulate the immune response to reduce inflammatory reaction and enhance the biocompatibility of the alloy. The specific influence of REEs differs with respect to individual element type and concentration.	-	-
Strontium	Promotes osteogenesis and angiogenesis and reduces bone resorption. The release of Sr^2+^ ions (with similar properties as Ca^2+^) can stimulate cell growth and differentiation.	0.3 g	0.17 mg
Silver	Induces antibacterial effect.	-	11–26 mg/L
Iron	Essential nutrient for life. However, the biocorrosion aspects needs to be carefully controlled. Can be metabolically regulated and stored.	4–5 g	5.0–17.6 g/L
Lithium	Assists in bone formation. Reduces kidney function and leads to central nervous system disorder.		2–4 ng/g
Manganese	Helps in bone formation and protects against infection.	12 mg	0.8 µg/L
Silicon	Cross-linking agent of connective tissue-based membrane structures. Necessary for growth as bone calcification.	18 mg	-
Aluminum	Releases Al^3+^ ions that can induce inflammatory response and oxidative stress in cells. Al is not generally recommended in biomedical alloys because of its potential neurotoxicity.	300 mg	2.1–4.8 µg/L
Nickel	Carcinogenic and toxic. Strong allergen that induces metal sensitivity.	10 mg	0.05–0.23 μg/L
Copper	Allergen. Trace element in cell. Induces cytotoxicity and inflammatory responses in cells due to the formation of Cu^2+^ ions.	200 mg	74–131 μmol/L
Tin	Carcinogenic. When used in larger amounts, leads to tin accumulation in lever.	3 mg	<0.1 µg/L
Zirconium	Toxic if used in higher concentrations.	250 mg	-
Polylactic acid	Biodegradable polymer. Metabolizes into lactic acid that is naturally occurring in the body		
Polyglycolic acid	Biodegradable polymer. Metabolizes into glycolic acid that is naturally occurring in the body	-	
Hydroxyapatite	Major component of human bones and teeth. Osteoconductive.	-	0.06–0.45 mmol/L
Tricalcium phosphate	Major component of human bones and teeth. Osteoconductive. Controlled degradation and increased inflammatory response than HA.	1–2 g/kg of body weight	0.13–1.38 mmol/L

**Table 4:** Key findings on clinical trials conducted on Mg alloys, as available from open literature.

Study	Type of Surgery	Type of Implant	Number of Patients	Average Age in Years	Clinical Follow-Up Timeline	Major Findings
Plaass et al. [100]	Symptomatic hallux valgus with indication for a Chevron-type osteotomy	MAGNEZIX^®^ CS 3.2 (Syntellix AG, Hannover, Germany)	40/44	45.5	6 weeks to 1 year	Magnesium-based implants degraded without any implant-directed inflammation reaction and possessed higher strengths than degradable polymer implants. Limitations: Specialized training needs for implant handing, different individual corrosion rates between patents
Biber et al. [101]	Intra-articular osteochondral fracture fixation	Cannulated MAGNEZIX compression screws	1	73	1 year	Uneventful consolidation of the osteochondral fracture of the elbow after MAGNEZIX screw fixation
Wichelhaus et al. [102]	Fracture reduction by palmar approach and retention to address scaphoid fracture.	Cannulated headless Magnezix screws	1	42	6 months	Despite good osseointegration properties, severe osteolysis occurred surrounding the Mg implants. Early degradation of Mg screws led to mechanical instability, which resulted in non-union and osteolysis.
Leonhardt et al. [103]	Fixation of displaced fractures of the condylar head	Magnezix CS 2.7 mm screw similar to the standard headless bone screw	5	73	-	Good reduction of the fractures and positioning of the screws No restriction in mandibular function within three months. No swelling associated with hydrogen gas or any other complications from the degradation of the material.
Biber et al. [104]	Chevron osteotomies, implant for lateral malleolar fracture fixation in an ankle fracture.	24 mm long MAGNEZIX^®^ CS 3.2	1	43	6 weeks	Uneventful healing accompanied by a radiolucency, which formed within six weeks postoperatively and had disappeared after 17 months. Findings inconsistent with former studies in terms of osteoconductive properties of Mg
Giganta et al. [105]	ARIF (arthroscopic reduction and internal fixation).	Magnezix	3	63–64	1–12 months	The implants were completely resorbed at the 6-month follow-up and replaced by newly formed bone at the 12-month follow-up.
Acar et al. [106]	Biplane chevron medial malleolar osteotomy (MMO) for osteochondral lesions of the talus (OLT)	(MAGNEZIX^®^ CS compression screws from Syntellix - Germany, compared against Ti64 headless compression screws (Acutrak^®^, Acumed, Hillsboro, OR, USA)	11	18–56	12–49 months	Results evaluated as per the American Orthopedic Foot and Ankle Society (AOFAS) scale and the visual analog scale (VAS) An improvement in the AOFAS scale and VAS points were recorded in both groups with no statistically significant difference between the groups. Complete union of the osteotomy was obtained in all patients. One patient in the Ti group required implant removal due to pain and irritation. There were no other significant complications in either group.
Choo et al. [107]	Forefoot reconstruction surgery with a scarf osteotomy	MAGNEZIX screw (Syntellix AG, Hannover, Germany).	24/69 (remaining Ti control group)	54.5 (21–71) years	12 months	Functional scores, radiological outcomes compared against a control group fixed with conventional titanium screws, and complication profile were recorded over 12 months.
Kose et al. [108]	Surgery to treat displaced medial malleolar fracture	Magnesium headless compression screws (MAGNEZIX^®^ CS, Syntellix AG, Hanover, Germany)	11		12–24 months	Mild radiolucency observed around the implants during the early postoperative period. This phenomenon does not cause any clinical symptom and did not adversely affect fracture healing.
Atkinson et al. [109]	Fixation of displacement 1st metatarsal osteotomies in the surgical management of hallux valgus by distal 1st metatarsal “short scarf” osteotomy	Magnesium compression screws (MAGNEZIX^®^ CS (3.2 mm diameter) compression screw) compared with titanium implants	25		12–30 month	PROM scores (Manchester-Oxford Foot Questionnaire (MOXFQ), Foot and Ankle Outcomes Instrument (FAOI), and the EQ-5D-3 L were recorded, pre-operatively. Magnesium and titanium patients showed similar patterns. Most patients reported a near full shoe comfort score, and EQ-5D-3 L scores were significantly improved in both patient groups (with most patients reporting a full score). No intra or post-operative complications. No problems encountered using the bioabsorbable screws.
Klauser et al. [110]	96 Youngswick and 4 Chevron-Osteotomies	A double-threaded compression screw (MAGNEZIX1 CS 3.2) vs. Fixos screws made of titanium alloy Ti 6Al-4V	95 Mg vs. 90 Ti	50.9 vs. 52.3	12.2 vs. 11.7 months	Mg screws were statistically non-inferior to the conventional titanium screws. No difference in mechanical stability, wound healing, or infection rate. Complete consolidation of the osteotomy was reported in all cases from radiologic, implant-specific findings.
Zhao et al. [111]	Treatment of necrosis	Pure Mg screws	1	17	2 years	CT images and radiographs clearly showed that the pure Mg screw was almost completely degraded. The functionality of hip assessed using Harris scores were found to be 37, 74, 83, and 86 for pre-operation, post-operation, one year, and two years, respectively.
Yu et al. [112]	Treatment of displaced femora	Combination of pure Mg implants with vascularized iliac grafting	19	22–45	8–24 months	Success rate was relatively higher up to 99.4% for the combination of internal fixation with osteotomy or vascularized iliac grafting.
Lee et al. [113]		Mg-5 wt%Ca-1 wt%Zn alloy	53	20	3	Long-term clinical study and systematic investigation of bone formation mechanism Complete bone healing occurred from biodegradable implant Simultaneous bone formation at the Mg alloy–bone interface allowed slow yet controlled degradation of Mg alloy implant that, within 1 year, was completely replaced by the new bone.
Windhagen et al. [114]	Hallux valgus surgery	Magnezix	26 (Either Ti or Mg, similar design)		6 months	No significant differences found in terms of the American Orthopedic Foot and Ankle Society (AOFAS) score for hallux, visual analog scale for pain assessment, or range of motion (ROM) of the first metatarsophalangeal joint (MTPJ). No foreign body reactions, osteolysis, or systemic inflammatory reactions were detected. The groups were not significantly different in terms of radiographic or laboratory results.
Zartner et al. [115,116]	Hybrid surgical procedure	Cardiovascular stent for balloon angioplasty (Biotronik)	1	Newborn	5 months	The reperfusion persisted throughout the 4-month follow-up period during which the gradual degradation process of the stent was completed and the same was clinically well-tolerated. The pathological and histological findings showed show minimal alteration of the vessel wall and an increase of the arterial diameter after stent degradation.
McMahon et al. [117]	Coronary Intervention	Cardiovascular stent for balloon angioplasty (Biotronik)	1	Newborn	4 months	Significant restenosis after 4 months of stent placement.
Schranz et al. [118]	Coronary Intervention	Cardiovascular stent for balloon angioplasty (Biotronik)	1	Newborn	3 months	While the immediate result was convincing and the baby was discharged, VSD closure with a patch augmentation of the previously stented aortic segment had to be performed after 3 months because of excess left to right shunting. The follow up angiography revealed the reduction of the lumen due to the degradation process of the stent, which not only allowed the operated vessel to backslide into its previous course, but even necessitated the implantation of a second stent. Despite the use of two metal stents, Mg was not detected in the serum of the patient and the residual Mg metal struts without stability forces were found, making the stented vessel segment flexible without influencing the surgical patch augmentation.
Maeng et al. [119]	Coronary Intervention	Cardiovascular stent for balloon angioplasty (Biotronik) compared with traditional stents	AMS (n = 11), sirolimus-eluting stents (Cypher; n = 11) and bare-metal stents (BMS; n = 9)	-	90 days	Neointima formation was measured by histomorphometry at 90 days. Vascular remodeling, defined as change in external elastic membrane area from index intervention to follow-up, was assessed by serial intravascular ultrasound (IVUS). Coronary implantation of absorbable magnesium stents, compared to two non-absorbable stents, was associated with the smallest lumen area at 3-month follow-up because of negative vascular remodeling. Neointima formation was smallest in the AMS group (*p* < 0.05 for both histomorphometry and IVUS).
Erbel et al. and Waksman et al. [120,121]	Coronary Intervention	Cardiovascular stent for balloon angioplasty (Biotronik)	63 patients, (44 men)	61.3	4, 6 and 12 months	No myocardial infarction, subacute or late thrombosis, or death occurred. Angiography at 4 months showed an increased diameter stenosis of 48·4, only small remnants of the original struts were visible and well embedded into the intima. Eight patients who did not require repeat revascularization at 4 months underwent late angiographic and IVUS follow-up from 12 to 28 months. Mg stents can be safely degraded after 4 months, while attempts are being recommended for prolonged degradation and drug elution.
Haude et al. [122]	Coronary Intervention	Balloon-expandable, paclitaxel-eluting scaffold (Biotronik) in symptomatic patients with de-novo coronary lesions.	46	-	1, 6, 12, 24 and 36 months	Results showed feasibility, a good safety profile, and promising clinical and angiographic performance results up to 12 months. Overall, device and procedural success was 100%. No cardiac death or scaffold thrombosis. In total, 2 of 46 (4%) patients had target lesion failure at 6 months, which rose to 3 of 43 (7%) at 12 months.
Haude et al. [123]	Coronary Intervention	Balloon-expandable, paclitaxel-eluting scaffold (Biotronik)	123	-	1, 6, 12, 24, and 36 months	At 6 months, mean in-segment late lumen loss was 0.27 mm 6.24 mm^2^ scaffold area left at 6 months No intraluminal mass or definite /probable scaffold thrombosis detected. Target lesion failure occurred in four patients: one died from cardiac death, one had periprocedural myocardial infarction, and two needed clinically driven target lesion revascularization.
Haude et al. [124]	Coronary Intervention	Cardiovascular stent for balloon angioplasty (Biotronik)	116	-	1, 6, 12 months and annually thereafter until 5 years	At six months, the in-scaffold late lumen loss was 0.21 mm. 7.58 mm^2^ scaffold area present after 6 months. Struts were embedded in the vessel wall and were already hardly discernible at six months. Target lesion failure occurred in one (0.9%) patient, although no definite or probable scaffold thrombosis or myocardial infarction was observed.

**Table 5:** Examples of commercially available magnesium implants.

Company	Country of Origin	Applications	Implant Type	Unique Features	Year Available	Website
Aap Implantate AG	Germany	Orthopedic	LOQTEQ^®^ plating systems, and cannulated screws	Biodegradable, promotes bone healing, reduces risk of inflammation and infection, custom design available	2011	https://www.aap.de/en/ accessed on 22 April 2023
Synthes	Switzerland	Orthopedic	Milagro^®^ screws, plates, and wires	Mg–Y–RE–Zr alloy specifically designed for fractures of the distal radius	2011	https://www.synthes.com/ accessed on 22 April 2023
Biotronik	Germany	Cardiovascular	Magmaris^®^ cardiovascular stents	Uses SynerMag^®^ material from magnesium elektron	2012	https://www.magmaris.com/ accessed on 22 April 2023
Syntellix AG	Germany	Orthopedic	MAGNEZIX^®^ screws, nails, anchors, and pins	Designed to degrade over time and be replaced by natural bone, range of implants available for orthopedic applications	2013	https://www.syntellix.com/ accessed on 22 April 2023
MeKo Laser Material Processing	Germany	Orthopedic	RESOLOY^®^ screws, plates, and nails	Custom implant design and manufacturing available	2015	https://www.meko.de/en/ accessed on 22 April 2023
HCM Orthocare	India	Orthopedic	MagOrtho™ screws, plates, and rods	Coated with a bioactive substance that promotes tissue regeneration, reduces risk of inflammation, and promotes bone healing	2015	https://www.magortho.com/ accessed on 22 April 2023
Medprin Regenerative Medical Technologies	China	Cardiovascular	MagLumine™ cardiovascular stent	The stent has a unique design that promotes arterial healing and reduces the risk of restenosis.	2015	http://en.medprin.com.cn/ accessed on 22 April 2023
Medical magnesium	Germany	Orthopedic	Interference screws, compression screws, and anchor systems	mm.Technology	2015	https://www.medical-magnesium.com/en/ accessed on 22 April 2023
MAGNEZIT GROUP	Russia	Orthopedic and cardiovascular	Magnesium screws, plates, and pins for orthopedic applications, as well as magnesium stents for cardiovascular applications	Use high-strength biodegradable magnesium alloy	2013	https://magnezit.ru/en/ accessed on 22 April 2023

**Table 6:** Benefits and limitations of different processing methods applicable to Mg materials.

Technique	Benefits	Limitations
Liquid state processing		
Gravity Die casting	▪ Simple process ▪ Economical	▪ Difficult to produce intricate shapes ▪ Undesirable interfacial reaction products
Pressure Die Casting	▪ Suitable for complex parts and large quantity production and economical ▪ Effective dispersion of reinforcement ▪ Better dimensional accuracy	▪ Undesirable reaction products ▪ Damage to reinforcement
Squeeze Casting	▪ Suitable for complex parts ▪ Effective dispersion reinforcement ▪ Capable of using larger reinforcement quantity than stir casting (up to 40–50%) ▪ Economical for large quantity production	▪ Damage to reinforcement ▪ Clustering of reinforcement
Compocasting	▪ Suitable for mass production ▪ Suitable for larger reinforcement quantity (up to 30%) ▪ Economical	▪ Damage to reinforcement ▪ Clustering of reinforcement ▪ Undesirable brittle interfacial reaction products ▪ Difficult to produce complex shapes ▪ Unstable vortex ▪ Gas entrapment
Ultrasonic assisted Compocasting	Better dispersion of reinforcement	▪ Unstable vortex ▪ Gas entrapment ▪ Damage to reinforcement
Plasma Spray Deposition	Finer microstructure due to faster solidification rates	▪ Unsuitable for complex and intricate shapes ▪ Expensive due to the use of gases ▪ Porosity
Disintegrated Melt Deposition	▪ Combine the benefits of stir casting and spray processing ▪ Flexible process in terms of reinforcement types and volume fractions ▪ Effective distribution of reinforcement ▪ Lesser chance for segregation ▪ Finer grain structure due to faster cooling rates	▪ Not suitable for intricate shapes ▪ Use of gases
Laser Energy Deposition	▪ Can fabricate complex shapes and structures ▪ High accuracy and precision	▪ Limited to small-scale production ▪ Surface roughness of the final product can be a challenge ▪ Possible defects, such as porosity and cracking, due to the thermal stresses generated by the process.
Solid State Processing		
Simple Blend-Press-Sinter	▪ Simple and economical processes ▪ Lesser interfacial reaction	▪ Not suitable for complex shapes ▪ Poor dispersion of reinforcement ▪ Chances of contamination due to binders
Microwave Sintering	▪ Energy efficient ▪ Faster process	▪ Segregation of reinforcement when used in larger amounts
Mechanical Alloying	▪ Suitable for different alloys including non-equilibrium alloys ▪ High strength, Strengthening due to high dislocation density ▪ Effective dispersion of reinforcement	▪ Not suitable for complex parts ▪ Not suitable for mass production ▪ Increased reactivity of powder materials
Spark plasma sintering	▪ Simple and rapid process	▪ Damage to reinforcement ▪ Unable to do complex shapes ▪ Energy intensive
Friction Stir Processing	▪ Solid-state methods without melting ▪ Relatively easy to control the process ▪ Effective dispersion of reinforcement by controlling the FSP parameters	▪ Process efficiency uncertain
Direct Ink Writing	▪ Ability to produce complex geometries and structures ▪ High control over material placement and layer thickness ▪ Ability to vary material properties by adjusting the ink formulation ▪ High-throughput production compared to other additive manufacturing techniques	▪ Limited to small-scale production ▪ Possible material shrinkage and deformation during the drying process ▪ Printing resolution and accuracy may be lower compared to other additive manufacturing techniques.
Ink Jet Printing	▪ Can produce complex shapes and structures with high control over material placement and layer thickness ▪ Enables the use of low-cost raw materials and can produce parts with high resolution and accuracy ▪ High-throughput manufacturing technique	▪ Limited material range due to ink formulation constraints ▪ Surface roughness and lower printing resolution/accuracy can be a challenge ▪ Mechanical properties of final product can be limited due to poor resolution and possible defects such as porosity.
Binder Jetting	▪ Can produce complex shapes and structures with high accuracy and precision ▪ High-throughput manufacturing technique ▪ No need for support structures during printing ▪ Cost effective and suitable for mass production	▪ Surface finish may be rough, requiring additional finishing steps ▪ Limited mechanical properties compared to conventionally manufactured parts due to the presence of binder material ▪ Material properties can be affected by the binder material used
Laser Powder Bed Fusion	▪ Produce complex shapes and structures with high accuracy and precision ▪ High throughput manufacturing technique ▪ Lesser material wastage ▪ Can produce parts with good surface finish and mechanical properties	▪ Part size is limited by the build envelope of the equipment ▪ The build process can be time-consuming ▪ Post-processing steps may be required for some applications, such as stress relief or additional finishing ▪ Material properties can be affected by the build parameters used ▪ The process can produce residual stresses and distortion in the final product

## Data Availability

Not applicable.
